# A comparative characterization of the circulating miRNome in whole blood and serum of HCC patients

**DOI:** 10.1038/s41598-019-44580-x

**Published:** 2019-06-04

**Authors:** Devis Pascut, Helena Krmac, Francesca Gilardi, Riccardo Patti, Raffaella Calligaris, Lory Saveria Crocè, Claudio Tiribelli

**Affiliations:** 1 Fondazione Italiana Fegato - ONLUS, Area Science Park, ss14, km163.5, 34149 Trieste, Italy; 2 Scuola Internazionale Superiore di Studi Avanzati - via Bonomea, 265 – 34136 Trieste, Italy; 3 Department of Medical Sciences, University of Trieste, Trieste, Italy; 4 Clinica Patologie Fegato, Azienda Sanitaria Universitaria Integrata di Trieste (ASUITS), Via Giovanni Sai 7, Trieste, Italy

**Keywords:** Tumour biomarkers, miRNAs

## Abstract

miRNAs are considered promising non-invasive biomarkers. Serum represents the major source of biomarkers, being readily accessible for many analytical tests. Recently, whole blood has drawn increasing interest in biomarker studies due to the presence of cancer-interacting cells and circulating cancer cells. Although Hepatocellular Carcinoma (HCC) is the seventh most frequent cancer worldwide, fragmented information exists regarding the miRNome characterization in blood and serum. We profiled the circulatory miRNome of paired serum and blood samples from 20 HCC patients, identifying 274 miRNA expressed in serum and 670 in blood, most of them still uncharacterized. 157 miRNA significantly differ between the two biofluids with 28 exclusively expressed in serum. Six miRNA clusters significantly characterize the two compartments, with the cluster containing miR-4484, miR-1281, miR-3178, miR-3613-3p, miR-4532, miR-4668-5p, miR-1825, miR-4487, miR-455-3p, miR-940 having the highest average expression in serum compared to blood. The ontological analysis revealed a role of these miRNAs in cancer progression, vascular invasion and cancer immune surveillance thought the regulation of DUSP1, PD-L1 and MUC1. Taken together, these results provide the most comprehensive contribution to date towards a complete miRNome profile of blood and serum for HCC patients. We show a consistent portion of circulatory miRNAs being still unknown.

## Introduction

Since their discovery in biofluids microRNAs (miRNAs) have drawn increasing interest in their use as circulatory biomarkers. These 21–23 short non-coding RNAs, responsible of the regulation gene expression in cells^1^, represent a class of promising non-invasive biomarker candidates as relatively stable to repeated freeze-thaw cycles, temperature and pH variations and prolonged storage^2^. More important, they are readily accessible in a wide range of biological samples, such as urine, saliva, blood, serum and other^3^.

Circulating miRNA profiles have been associated to different diseases^4^ but the most striking application regards several aspects of malignancies. Despite Hepatocellular Carcinoma (HCC) represents one of the most frequent cancers worldwide, ranking as the second cause of cancer-related death^5^, a comprehensive characterization of the circulatory microRNA profiling is still missing. Actually 2588 mature human miRNAs are annotated on miRBase 21 (http://www.mirbase.org); however only a small fraction has been studied and characterized in HCC. Most of the investigations of miRNA as biomarkers consider serum or plasma as the most readily available and promising reservoir of miRNAs. Recently, whole blood have been considered as an alternative source of miRNAs^6^ derived from blood cells such as neutrophils, monocytes, mature red blood cells^7^, regulatory T cells, recruited to the tumour site^8^, and cancer circulating cells. Since the role of these cells in cancer diseases, whole blood miRNAs became an attractive source of potential biomarkers during cancer pathogenesis.

Despite the increasing number of miRNA studies in HCC, a complete miRNome characterization of the biofluids is missing. In this study we performed an expression profiling of 1733 circulating miRNA from paired whole blood and serum samples. We believe that the fully characterization of these two compartments may provide important information on the miRNA species that characterize the two biofluids with relevant implications in the interpretation of future cancer biomarker studies.

## Materials and Methods

### Patients

Twenty consecutive patients referring to the Liver Center between 2012 and 2017 with HCC according to the EASL criteria were enrolled for the study. Samples were collected at the time of HCC diagnosis, before any oncological treatment. The clinical and demographic features of the groups are shown in Table S1.

All the patients provided written informed consent and patient anonymity has been preserved. Investigation was conducted according to the principles expressed in the Declaration of Helsinki. The study was approved by the regional ethical committee (Comitato Etico Regionale Unico FVG, No. 14/2012 ASUITS).

### Sample collection

Fasting paired whole blood and serum samples were collected at the same time from each patient during clinical visits. Peripheral blood (3 ml) was collected in Tempus™ Blood RNA Tube containing 6 mL Stabilizing Reagent (Thermo Fischer Scientific, USA) and subsequently frozen at −80 °C for long-term storage. Serum was collected in Vacuette^®^ serum separating tubes (Greiner Bio-One GmbH, Austria). Samples were centrifuged at 3500 rpm for 10 minutes, aliquoted and stored at −80 °C until further use.

### Assessment of hemolysis

The hemoglobin in serum was assessed with Beckman Coulter® DU®730 spectrophotometer using the Harboe Direct spectrophotometric method with Allen correction: Hb(g/l) = (167.2 × A_415_ − 83.6 × A_380_ − 83.6 × A_450_) × 1/1000 × 1/dilution in dH_2_O. The considered cut-off for serum was 0.020 g/L.

### RNA isolation and quality assessment

Total RNA was isolated from Tempus™ Blood Tubes using the MagMAX™ for Stabilized Blood Tubes RNA Isolation Kit (Thermo Fischer Scientific, USA) following the manufacturer protocol. Small RNAs, were isolated from 300 uL of serum using the miRCURY™ RNA Isolation Kits (Exiqon, Vedbaek Denmark) and quantified with the Qubit microRNA Assay Kit (Thermo Fischer Scientific, USA). The quality of the total RNA and miRNAs extracted was assessed with the Agilent RNA 6000 Nano Kit (agilent Technologies, USA) and Agilent Small RNA kit (Agilent Technologies, USA) respectively, by using the 2100 Bioanalyzer Instrument (Agilent Technologies, USA). Samples with RIN less than 6 were discarded from the subsequent profiling experiments.

### Microarray profiling

Small RNAs were extracted from whole blood and serum and independently profiled on the Affymetrix microRNA arrays containing 1,734 human mature miRNA probe sets. 130ng of RNA were labelled with the FlashTag™ Biotin HSR RNA Labeling Kit (Affymetrix®, Thermo Fischer Scientific, USA) and hybridized on Genechip miRNA 3.0 (Thermo Fischer Scientific, USA). Array cartridges were processed on an Affymetrix Fluidic Station 450 and scanned on an Affymetrix GeneChip 3000 7G. The robust Multichip Analysis (RMA) algorithm was used to derive CEL file probe-level hybridization intensities at the gene expression levels.

### *In silico* data analysis

We search for the miRNA expression patterns in the miRmine database (http://guanlab.ccmb.med.umich.edu/mirmine/index.html) that collects the expression profiles from different publicly available miRNA-seq datasets. Validated miRNA targets were downloaded from http://mirtarbase.mbc.nctu.edu.tw/php/index.php. GOrilla (http://cbl-gorilla.cs.technion.ac.il), was used to determine pathway and GO terms enrichment of the miRNA validated targets. The search was relative to a background set of approximately 14,800 human genes that could be associated with any miRNA from MiRTarBase. Enrichment results were visualized as “TreeMaps” generated in REViGO (http://revigo.irb.hr/). MiRNA target networks were built by using Tidyverse (version 1.2.1) and Igraph (version 1.1.2) packages in R environment (RStudio Team (2015). RStudio: Integrated Development for R. RStudio, Inc., Boston, MA (http://www.rstudio.com/). The StarBase v.3.0 database^9^, a Clip-Seq based miRNA expression dataset, generated by The Cancer Genome Atlas (TCGA) repository was used to search for differently expressed miRNAs in HCC tissues.

### Data analysis and statistical methods

Gene expression differences between paired blood and serum were calculated with the Affymetrix transcriptome analysis console using paired one-way analysis of variance (ANOVA) test. Multiple testing correction was performed with the Benjamini-Hochberg method and false discovery rate (FDR), corrected p-values were calculated. The relationship between expression levels of each miRNA within the family was analyzed using Pearson correlation. Correlation matrices were generated with R studio software by using and the Hmisc (version 4.1-1) Performance Analytics (version 1.4.3541) and Corrplot (version 0.84) packages.

## Results and Discussion

### Profiling of total blood and serum identifies different patterns of microRNAs

We profiled the miRNome of 20 paired total blood and non-hemolized serum samples from HCC patients though the Affymetrix Genechip® mirna 3.0 array build on miRBase release 20.

According to the Absent/Present calling of the Affymetrix algorithm, we divided the circulatory miRNome in five quantiles based on their presence in the analyzed population. We considered the miRNAs in the first quantile as not expressed miRNAs, since they were detectable in less than five subjects. We found 1459 out of the 1734 mature miRNA not expressed in serum, 99 were expressed in the 2^nd^ quantile of the population (considered as rare miRNAs), 53 and 43 in the 3^rd^ and 4^th^ quantiles, respectively, and 79 in the 5^th^ quantile (Table 1).

**Table 1:** MiRNA expression in blood and serum of HCC patients.

	**SERUM**					
		**2** ^**nd**^ **quantile**	**3** ^**rd**^ **quantile**	**4** ^**th**^ **quantile**	**5** ^**th**^ **quantile**	**Not expressed**
BLOOD	2^nd^ quantile	miR-4490, miR-4423-3p, miR-4439, miR-297, miR-3152-3p, miR-4657, miR-200b-3p, miR-2277-5p, miR-1972, miR-335-5p, miR-4797-5p, miR-3927-3p	miR-4725-5p, miR-32-5p, miR-548ai	mir-548aa	none	miR-100-3p, miR-101-3p, miR-1245b-3p, miR-1257, miR-1278, miR-1284, miR-1296-5p, miR-140-5p, miR-146b-3p, miR-154-5p, miR-188-3p, miR-214-3p, miR-21-3p, miR-299-3p, miR-29b-3p, miR-29b-1-5p, miR-29c-3p, miR-301a-3p, miR-30b-3p, miR-30e-3p, miR-3129-5p, miR-3140-3p, miR-3146, miR-3150a-5p, miR-3151-5p, miR-3156-5p, miR-3173-5p, miR-325, miR-338-5p, miR-34c-3p, miR-3607-5p, miR-3617-5p, miR-3622b-3p, miR-3650, miR-3667-3p, miR-3675-3p, miR-3689c, miR-370-3p, miR-373-5p, miR-374a-5p, miR-374b-5p, miR-379-5p, miR-3918, miR-3925-5p, miR-3934-5p, miR-4257, miR-4266, miR-4272, miR-4294, miR-4326, miR-4428, miR-4434, miR-4435, miR-4444, miR-4465, miR-4478, miR-4511, miR-4540, miR-4635, miR-4653-3p, miR-4659a-3p, miR-4660, miR-4662a-3p, miR-4698, miR-4703-3p, miR-4705, miR-4720-3p, miR-4721, miR-4725-3p, miR-4763-5p, miR-4766-5p, miR-4774-5p, miR-4778-5p, miR-4795-5p, miR-485-5p, miR-493-5p, miR-509-3p, miR-548ab, miR-554, miR-557, miR-595, miR-598-3p, miR-616-5p, miR-623, miR-765, miR-922
	3^rd^ quantile	miR-3942-5p, miR-3128, miR-19a-3p, miR-4717-3p, miR-4708-3p, miR-4445-3p, miR-548i, miR-603	miR-3910, miR-1323, miR-16-2-3p, miR-4720-5p	miR-1272, miR-548z, miR-4474-3p, miR-628-5p	mir-940	let-7i-3p, miR-1-3p, miR-101-5p, miR-103a-2-5p, miR-1179, miR-1208, miR-1229-3p, miR-1244, miR-1273e, miR-1289, miR-130a-5p, miR-1537-3p, miR-184, miR-1912, miR-193a-3p, miR-194-3p, miR-196b-5p, miR-221-5p, miR-22-5p, miR-23b-5p, miR-24-2-5p, miR-26b-3p, miR-27b-5p, miR-296-3p, miR-219b-5p, miR-3064-3p, miR-3164, miR-3174, miR-3180-3p, miR-32-3p, miR-330-3p, miR-3529-5p, miR-3651, miR-3688-3p, miR-3689b-3p, miR-3976, miR-4288, miR-4323, miR-433-3p, miR-4419b, miR-4422, miR-4442, miR-4448, miR-4455, miR-4461, miR-4472, miR-4506, miR-4514, miR-451b, miR-4521, miR-4525, miR-4538, miR-4654, miR-4747-5p, miR-4748, miR-4755-5p, miR-4784, miR-4800-5p, miR-485-3p, miR-497-5p, miR-500b-5p, miR-504-5p, miR-512-3p, miR-543, miR-548ah-5p, miR-548k, miR-569, miR-640, miR-654-3p, miR-924, miR-96-5p
	4^th^ quantile	miR-664a-3p, miR-3622a-3p, miR-143-3p, miR-551b-5p, miR-4440, miR-1255a	miR-642b-3p, miR-4727-3p, miR-483-5p, miR-3201, miR-675-3p, miR-2053, miR-4742-5p	miR-3157-3p, miR-191-3p, miR-1238-3p, miR-548aj-3p, miR-548ae-3p, miR-548 × -3p, miR-1246	miR-1184, miR-4706, miR-1263	miR-103b, miR-127-3p, miR-1287-5p, miR-129-2-3p, miR-1299, miR-134-5p, miR-181d-5p, miR-1827, miR-186-5p, miR-188-5p, miR-192-3p, miR-199a-3p, miR-199b-3p, miR-206, miR-208b-3p, miR-20b-3p, miR-21-5p, miR-2278, miR-3064-5p, miR-3136-5p, miR-3138, miR-3147, miR-3150b-3p, miR-3157-5p, miR-3177-3p, miR-3179, miR-3192-5p, miR-326, miR-330-5p, miR-34a-5p, miR-34b-3p, miR-3668, miR-3680-3p, miR-371a-5p, miR-382-5p, miR-3909, miR-3928-3p, miR-4309, miR-4312, miR-432-5p, miR-4321, miR-4419a, miR-4458, miR-4485-3p, miR-4498, miR-4499, miR-4526, miR-4535, miR-454-3p, miR-4646-5p, miR-4667-5p, miR-4672, miR-4685-5p, miR-4697-3p, miR-4723-5p, miR-4738-3p, miR-4753-5p, miR-4788, miR-4789-3p, miR-487b-3p, miR-502-5p, miR-514b-5p, miR-542-5p, miR-556-3p, miR-576-5p, miR-616-3p, miR-624-5p, miR-641, miR-665, miR-671-3p, miR-7-5p, miR-7-1-3p, miR-769-3p, miR-874-3p, miR-942-5p
	5^th^ quantile	let-7a-5p, miR-1225-5p, miR-125b-5p, miR-1273f, miR-1307-3p, miR-130a-3p, miR-145-5p, miR-150-5p, miR-150-3p, miR-151a-5p, miR-155-5p, miR-15a-5p, miR-15b-5p, miR-181b-5p, miR-18a-5p, miR-1909-5p, miR-20b-5p, miR-222-3p, miR-23c, miR-26a-5p, miR-27a-3p, miR-29a-3p, miR-30a-5p,miR-3162-5p, miR-342-3p, miR-361-5p, miR-3648, miR-3679-5p, miR-378c, miR-378f, miR-378i, miR-3935, miR-4286, miR-4306, miR-4430, miR-4481, miR-4492, miR-4539, miR-4749-5p, miR-4791, miR-4800-3p, miR-486-3p, miR-491-5p, miR-502-3p, miR-503-5p, miR-532-5p, miR-550a-3p, miR-574-5p, miR-629-3p, miR-885-3p, miR-885-5p, miR-939-5p	let-7d-5p, let-7i-5p, miR-103a-3p, miR-107, miR-1224-5p, miR-126-3p, miR-1268b, miR-1275, miR-130b-3p, miR-140-3p, miR-146a-5p, miR-17-5p, miR-181a-5p, miR-191-5p, miR-1910-5p, miR-193a-5p, miR-197-3p, miR-20a-5p, miR-221-3p, miR-223-3p, miR-23b-3p, miR-24-3p, miR-30d-5p, miR-3124-5p, miR-3135b, miR-320e, miR-3621, miR-3646, miR-371b-5p, miR-409-3p, miR-4253, miR-4459, miR-4486, miR-4741, miR-574-3p,	let-7c-5p, miR-106a-5p, miR-106b-5p, miR-1207-5p, miR-1260a, miR-1260b, miR-1268a, miR-151a-3p, miR-16-5p, miR-19b-3p, miR-22-3p, miR-23a-3p, miR-25-3p, miR-3141, miR-378a-3p, miR-423-5p, miR-425-5p, miR-425-3p, miR-4433a-3p, miR-4454, miR-4690-5p, miR-4739, miR-4758-5p, miR-498, miR-584-5p, miR-92b-3p, miR-93-5p, miR-933	let-7b-5p, let-7b-3p, let-7f-1-3p, miR-122-5p, miR-1228-3p, miR-1228-5p, miR-1280, miR-1281, miR-1469, miR-149-3p, miR-1587, miR-1825, miR-185-5p, miR-1908-5p, miR-1915-3p, miR-2861, miR-3178, miR-3185, miR-3196, miR-320a, miR-320b, miR-320c, miR-320d, miR-3613-3p, miR-3613-5p, miR-3619-5p, miR-3656, miR-3665, miR-378h, miR-3921, miR-3940-5p, miR-3960, miR-4270, miR-4274, miR-4281, miR-4310, miR-4429, miR-4443, miR-4463, miR-4466, miR-4467, miR-4484, miR-4487, miR-4488, miR-4497, miR-4505, miR-4507, miR-4508, miR-451a, miR-4516, miR-4529-3p, miR-4530, miR-4532, miR-455-3p, miR-4649-5p, miR-4651, miR-4668-5p, miR-4674, miR-4687-3p, miR-4689, miR-4695-5p, miR-4707-5p, miR-4710, miR-4734, miR-4745-5p, miR-4763-3p, miR-4787-5p, miR-486-5p, miR-548a-3p, miR-548ac, miR-638, miR-663a, miR-762, miR-877-5p, miR-92a-3p	let-7d-3p, let-7e-5p, let-7f-5p, let-7g-5p, miR-100-5p, miR-106b-3p, miR-1180-3p, miR-1202, miR-1226-3p, miR-1231, miR-1254, miR-1255b-5p, miR-125a-5p, miR-1270, miR-1271-5p, miR-1273c, miR-128-3p, miR-1285-3p, miR-1292-5p, miR-1294, miR-129-5p, miR-1301-3p, miR-1303, miR-1304-5p, miR-1306-3p, miR-130b-5p, miR-132-3p, miR-139-5p, miR-146b-5p, miR-148a-3p, miR-148b-3p, miR-151b, miR-152-3p, miR-17-3p, miR-181a-2-3p, miR-181c-5p, miR-182-5p, miR-183-5p, miR-183-3p, miR-185-3p, miR-18a-3p, miR-18b-5p, miR-1909-5p, miR-192-5p, miR-194-5p, miR-195-5p, miR-1973, miR-1976, miR-199a-5p, miR-200c-3p, miR-202-3p, miR-210-3p, miR-2110, miR-2276-3p, miR-1281-3p, miR-2392, miR-23a-5p, miR-25-5p, miR-26b-5p, miR-27b-3p, miR-28-3p, miR-28-5p, miR-29b-2-5p, miR-29c-5p, miR-3074-5p, miR-30b-5p, miR-30c-5p, miR-30c-1-3p, miR-30e-5p, mir-31-5p, miR-3127-5p, miR-3129-3p, miR-3130-5p, miR-3149, miR-3158-3p, miR-3158-5p, miR-3163, miR-3173-3p, miR-3180, miR-3187-3p, miR-3188, miR-3195, miR-3200-3p, miR-3200-5p, miR-324-3p, miR-324-5p, miR-328-3p, miR-331-3p, miR-331-5p, miR-337-5p, miR-339-3p, miR-339-5p, miR-342-5p, miR-345-5p, miR-346, miR-3605-3p, miR-3605-5p, miR-3609, miR-361-3p, miR-3615, miR-3619-3p, miR-3620-3p, miR-362-5p, miR-363-3p, miR-363-5p, miR-3652, miR-3667-5p, miR-3682-3p, miR-3691-5p, miR-378b, miR-378d, miR-378e, miR-378g, miR-378a-5p, miR-3911, miR-3936, miR-3937, miR-3940-3p, miR-3944-5p, miR-421, miR-422a, miR-423-3p, miR-424-3p, miR-4271, miR-4284, miR-4298, miR-4299, miR-4317, miR-4322, miR-4327, miR-4436b-5p, miR-4447, miR-4462, miR-449b-3p, miR-4500, miR-4510, miR-4513, miR-4534, miR-4634, miR-4640-5p, miR-4655-5p, miR-4656, miR-4665-5p, miR-4669, miR-4676-5p, miR-4685-3p, miR-4688, miR-4695-3p, miR-4701-3p, miR-4708-5p, miR-4728-5p, miR-4732-3p, miR-4732-5p, miR-4736, miR-4743-5p, miR-4750-5p, miR-484, miR-494-3p, miR-500a-5p, miR-500a-3p, miR-501-3p, miR-501-5p, miR-505-3p, miR-505-5p, miR-532-3p, miR-548q, miR-550a-5p, miR-550b-3p, miR-572, miR-575, miR-589-5p, miR-589-3p, miR-610, miR-625-5p, miR-628-3p, miR-629-5p, miR-636, miR-652-3p, miR-660-5p, miR-664a-5p, miR-671-5p, miR-720, miR-744-5p, miR-766-3p, miR-769-5p, miR-92b-5p, miR-93-3p, miR-941, miR-943, miR-98-5p, miR-99a-5p, miR-99b-5p
	Not expressed	miR-202-5p, miR-2116-3p, miR-3121-3p, miR-3153, miR-323b-3p, miR-337-3p, miR-3663-3p, miR-3942-3p, miR-4290, miR-4646-3p, miR-4652-3p, miR-4701-5p, miR-4722-3p, miR-4789-5p, miR-4793-3p, miR-548u, miR-559, miR-570-3p, miR-602, miR-635, miR-877-3p	miR-1234-3p, miR-4452, miR-450b-5p, miR-563.	miR-4258, miR-3197, miR-3162-3p	None	All the remaining

In blood 1064 miRNAs were undetectable, 102 were in the 2^nd^ quantile while 382 were present in the 5^th^ quantile of the population (Table 1). Among the four quantiles of expressed miRNAs in the two biofluids, 28 serum miRNAs were not expressed in blood, suggesting a pure extra-blood origin; most of them belong to the 2^nd^ serum quantile representing the rarely detected miRNAs. MiR-4258, miR-3197 and miR-3162-3p are abundantly expressed only in serum of HCC patients. Seventeen miRNAs expressed in serum were rarely detected in blood (2^nd^ blood quantile), while 1035 were undetectable in both biofluids. On the contrary, 424 miRNAs were uniquely detected in blood (Table 1).

**Figure 1:**
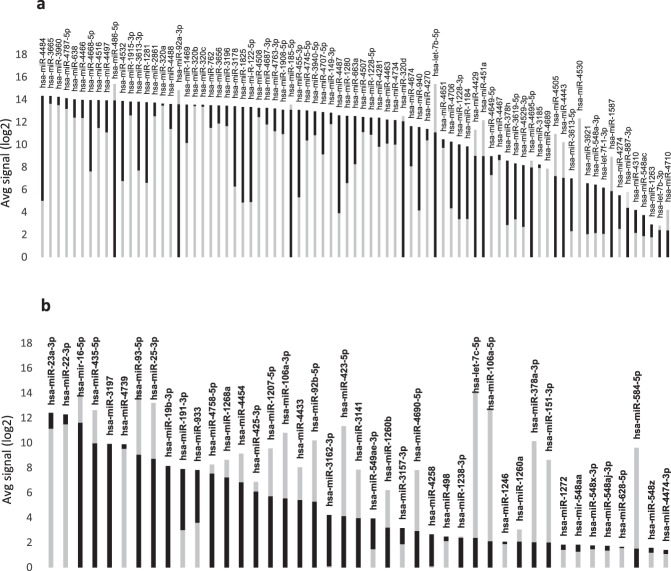
MiRNA average expression (log2) determined by miRNA microarray. The black bars correspond to the expression in serum while the grey ones correspond to the expression in whole blood. (**a**) MiRNA expressed in the 5^th^ and (**b**) 4^th^ serum quantile.

Considering the miRNAs log2 average signal intensities the 5^th^ serum group contains the miRNAs with the highest expression in serum and the higher expression compared to blood, with the exception of mir-486-5p, -92a-3p, -185-5p,-320d, -let7b-3p, -4429,- 451a; -4505, -4443, -4530, -1587, -877-5p, -4710. Mir-4689 has the same expression in both biofluids (Fig. 1a).

**Figure 2:**
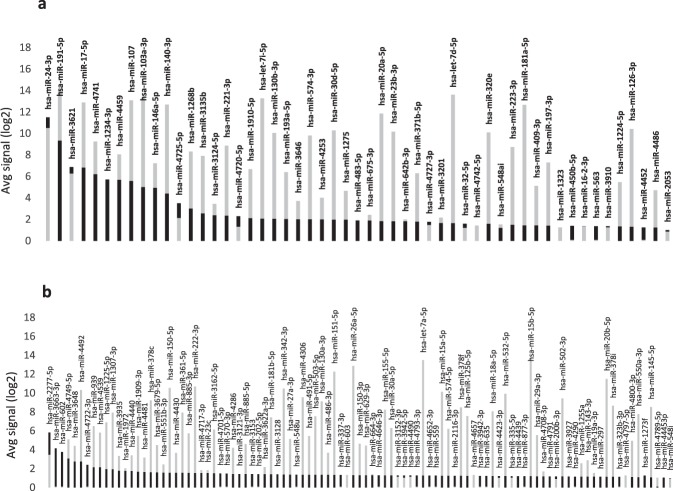
MiRNA average expression (log2) determined by miRNA microarray. The black bars correspond to the expression in serum while the grey ones correspond to the expression in whole blood. (**a**) MiRNA expressed in the 3^rd^ and (**b**) 2^nd^ serum quantile.

The serum 4th quantile has a more skewed distribution of the miRNA expression levels. The 50% of the miRNAs have a higher expression in blood, mir-let7c-5p, -106a-5p, -378-3p, -151a-3p and miR-584-5p have the highest blood expression compared to serum with 8.64 -14.16 (min-max) average expression in blood *vs*. 1.59-2.37 (min-max) average expression in serum (Fig. 1b). MiR-4258, miR-3197 and miR-3162-3p were not detected in blood but have a high expression level in serum suggesting a pure extra-blood origin. Interestingly, very limited data existing link these miRNAs to cancer^10,11^. Mir-4258 was associated to chemo-resistant breast cancer cell lines and FFPE tissues^10^, while mir-3197 was up-regulated in colorectal liver metastases^11^. No or limited evidence exists about the expression of these miRNAs in HCC, offering new opportunities for future explorative studies in clinical specimens. Twelve out of fifty-three miRNAs have a higher expression in serum compared to blood in the third serum group. MiR-4452, -1234-3p, -563, and miR-450b-5p were undetectable in blood. In this group, 29 miRNAs have an average log2 signal intensity less than 2. Mir-24-3p, -1234-3p, -3621, and miR-4720 -5p are the only miRNAs with an average log2 signal intensity greater than 2 and with the higher expression in serum (Fig. 2a).

The miRNAs in the 2^nd^ quantile have the lowest average log2 signal intensities in serum. We defined them as rare miRNAs since they are present in less than the 40% of the screened population. Twenty-one of them were not expressed in blood. Mir-200b-3p, -297, -335-5p, -1972, -2277-5p, -3152-3p, -3927-3p, -4439, -4490, -4657, -4797-5p are also low expressed and rare miRNA in blood with the only exception of miR-2277-5p that has an average expression of 5.84 (Fig. 2b).

Fifty-two highly blood expressed miRNAs belonging to 5^th^ blood quantile are also present in this group, with the exception of miR-23c, -1273f, -4791, that have a low average expression.

### Differentially expressed miRNAs in whole blood and serum

We then compared the expression of the shared miRNA between the two biofluids, identifying 157 miRNA significantly differently expressed between total blood and serum (Anova p < 0.05). We filtered the miRNAs, identifying a total of 28 highly expressed miRNAs in serum, with a ≥10-fold higher levels compare to total blood, all belonging to the serum 5^th^ quantile, except miR-933, and miR-191-3p (Table S2). Among them, the liver specific miRNA miR-122-5p shows more than 348 fold increase in serum. Several other miRNAs were already associated to cancer, such as miR-455-3p^12^, -933^13^, -940^14^, including HCC^13,15^ in which miR-455-3p and miR-940 were downregulated in tumor liver tissue, compared to the surrounding disease^13^ or normal livers^15^, while mir-933 was upregulated^13^. To our knowledge, no evidence exists about their presence and clinical significance in serum of HCC patients. Regarding the recently discovered miRNAs, the lack of experimental evidences linking them to cell pathways or specific diseases make difficult any speculation about their cell origin or clinical meaning.

Seventy-eight miRNAs have a ≥10-fold higher levels in blood than in serum. Among them, miR-150, -155, -223 were already known for their blood origin, being highly expressed in hematopoietic cells^16^ and mature blood cells. miR-223, together with miR-197, -574-3p and let-7a represent the previously identified myeloid miRNA signature^7^ with substantial differences in the expression between serum and total blood (Table S2). In addition, miR-150 is reported to be highly expressed in human lymphocytes^7^. Twenty-six miRNAs have ≤2 Fold of Change (FC) < 10 higher in total blood compared to serum, in this group the red blood cells (RBC) miR-451-3p; -92a-3p, -16-5p, -486-5p^7^ are highly expressed in both compartments. In plasma hemolytic samples their expression increase with the extent of the hemolysis suggesting the high impact of RBC in the circulatory miRNA profile^17^. The differences in the average expression level in serum of the RBC-derived and white-cells-derived miRNAs suggest a significant contribution from the erythrocytes even in normal conditions (non-hemolyzed samples). Twenty-seven miRNAs, mostly belonging to the serum 5^th^ quantile, have ≤2 FC < 10 higher expression in serum. Twenty-four miRNAs significantly differ with small changes in the two fluids (±1.1 ≤ FC < ±2). Sixty-three are expressed at comparable levels in both blood and serum.

Kirschner^17^ found a plasma miRNA signature influenced by hemolysis, including let7b-5p, miR-126-3p, -140-3p, -15a-5p, -15b-5p, -16-5p, -20a-5p, -20b-5p, -320a, -425-5p, -486-3p, -140-3p, -451a. Those miRNAs are overexpressed in blood compared to serum, furthermore miR-194-5p, -21-5p, -210-3p, -26b-5p, -324-3p, -331-5p, -454-3p, -484, -532-3p, 652-3p are exclusively found in blood in our samples. Interestingly, their suggested signature unaffected by haemolysis, including let-7a-5p, let7d-5p, miR-127-3p, -130b-3p, 143-3p, -146a-5p, -221-3p, -222-3p, -27b-3p, 324-5p, -338-5p, 339-5p, -744-5p is also overexpressed or exclusively expressed in our blood samples (Tables 1 and S2). Supporting our observations, Sangokoya^18^ reported that let7 family miRNAs 7a, 7b, 7c, 7d, 7f are highly expressed in RBC since their role in reticulocytes. In our samples, miR-15b-5p, let-7a-5p and let-7d-5p have the highest blood expression with 5728, 4767 and 3824 fold of change compared to serum, respectively. These miRNAs increase even in presence of mild hemolysis in our samples (Fig. S1). The group of miRNAs unaffected by hemolysis (green cluster, Fig. S1), confirms only the previously identified miR-143-3p, while, hemolysis has a major effect on serum miR-130a-3p, -130b-3p, 15a-5p, -18a-5p, -30a-5p, -320e, -4506, -532-5p, -584-5p, included in the red cluster (Fig. S1).

### Circulating miRNome cluster analysis

**Figure 3:**
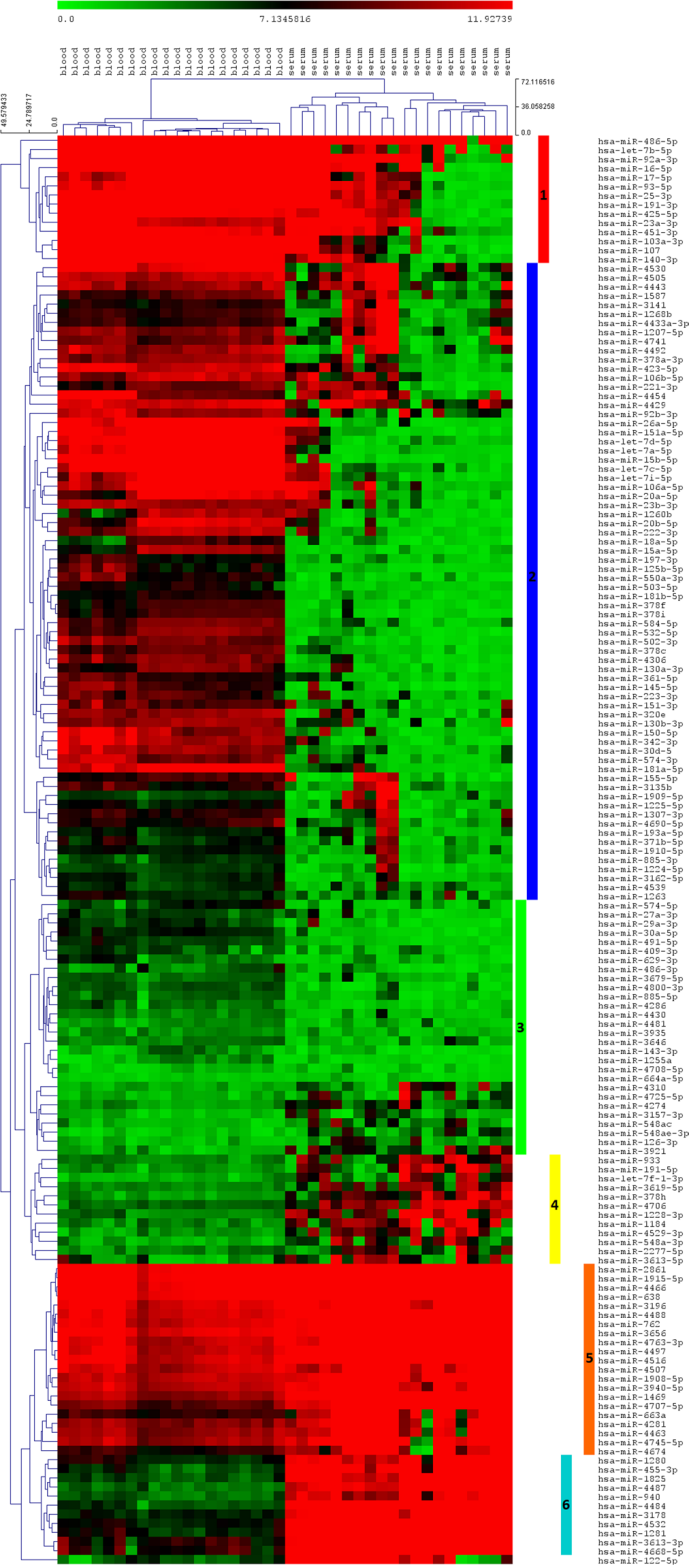
Heatmap with the pseudocolor scale underneath, of the differentially expressed miRNAs among the 40 samples. Unsupervised hierarchical clustering is used to order samples and miRNAs, the log2-transformed microarray signal was considered. The sample tree with optimized leaf-ordering is drawn using Euclidean distances and average linkages for cluster-to-cluster distance. The six major clusters are identified by different colors: Cluster 1 (red), cluster 2 (blue), cluster 3 (green), cluster 4 (yellow), cluster 5 (orange), cluster 6 (light blue).

The unsupervised hierarchical clustering using Euclidean distances correlation measures of the intensities values for the set of blood and serum shared miRNAs showed the expected clustering of the cases indicating presence of blood and serum-specific miRNA expression profiles (Fig. 3).

Six distinguishable clusters were identified among the expressed miRNA. Cluster 1 (red) includes miRNAs with high expression level in blood, and the 50% of the serum samples have miRNA expressions comparable to blood. These RNAs include miR-486-5p, let-7b-5p, miR-92a-3p, miR-103a-3p, miR-107, miR-140-3p, miR-16-5p, miR-451a, miR-23a-3p, miR-17-5p, miR-191-3p, miR-25-3p, miR-425-5p, miR-93-5p. These miRNAs are widely and highly expressed among different human tissues (Fig. S2) and regulate key cellular pathways, such as cell proliferation, cell death, kinase activities, aging, metabolic pathways, as evidenced by a validated target enrichment analysis performed with GOrilla (Fig. S3 and Supporting File 1). Those miRNAs have been considered as circulating biomarkers for multiple diseases^19^ including HCC^20^. However, they are highly expressed in a wide range of tissues, including blood cells. Therefore blood must be considered as a source of these miRNAs in case of improper sample collection^17^, causing a misinterpretation of the results in biomarker discovery studies.

Cluster 2 (blue) include miRNAs with high expression levels in blood and in some cases comparable expression in serum. This cluster includes a heterogenic profile of circulating miRNAs, involved in a wide variety of cellular pathways, some of them referring to blood-cells-related pathways: response to oxygen levels, regulation of kinase activity, regulation of immune system, hemopoiesis and regulation of apoptotic process (Fig. S4 and Supporting File 2).

Cluster 3 (green) includes the low expressed miRNAs in both serum and blood. Only four miRNAs of this cluster were previously studied: MiR-27a-3p, -29a-3p, -30a-5p, -143-3p. In HCC, miR-29a-3p, 30a-5p and 143-3p work as oncosuppressors^21–23^, while contrasting information exists about the oncogenic role of miR-27a, probably due to a different role of the two mature isoforms coming from the hairpin precursors^24,25^. Unfortunately, we were not able to identify the mature form studied in previous works in HCC.

Cluster 4,5 and 6 include miRNAs with higher expression in serum. The expression of miRNAs in Cluster 4 is highly variable, going from 5.89 FC of miR-2277-5p to 97 FC of miR-1228-3p. Very limited information regarding the cellular role of these miRNAs exist. MOAP1 (Modulator Of Apoptosis 1) and CSNK2A2 (Casein Kinase 2 Alpha 2)^26,27^ are the only validated targets for miR-1228-3p, with opposite effects on cancer behavior, leading to a still questionable role of this miRNA in cancer.

To cluster 5 (orange) belong high expressed miRNAs in both biofluids, although there is a clear difference between the two compartments. Among the 21 members of the cluster only 6 miRNA have experimentally validated target genes on miRTarBase (http://mirtarbase.mbc.nctu.edu.tw), (miR-638; -663a; -762; -1908-5p; -4516; 4674). The GO target enriched analysis evidenced the involvement of these miRNAs in key cell pathways such as cell cycle, cell migration, cell death regulation and drug response, often dysregulated in cancer (Fig. S5 and Supporting File 3). Since their high expression in both compartments, we hypothesize these miRNAs could represent a portion of miRNA widely released from body cells, although still missing information exists about tissue profiling on these miRNA (Fig. S6).

In cluster 6 (light blue), 10 miRNAs show high differences in the serum compared to blood samples, going from 68.61 FC, of miR-3613-3p, to 633.01 FC of miR-4484. To this cluster belong rather unknown miRNAs both in terms of function and clinical significance. The only validated targets listed in miRTarBase are DUSP1 (Dual Specificity Phosphatase 1) and CD274 for -miR-940 and MUC1 (Mucin 1) for miR-455-3p. Interestingly DUSP1 and CD274, also known as B7H1 (B7 Homolog 1) or PD-L1 (PD-L1 - Programmed Cell Death 1 Ligand 1), are involved in cancer progression and vascular invasion^28^, and cancer immune surveillance^29^. The importance of these two targets was reported in two recent publications in which a relevant role of these two targets was discovered in HCC^30,31^. DUSP1 was linked to HCC development and progression^31^, while the expression of PD-L1 in neoplastic or intra-tumoral inflammatory cells was related to tumor aggressiveness and response to treatments. Further evidence links the expression of miR-940 to a suppression of tumor invasion and migration^32^ and to apoptosis induction of cancer cells^33^. For the remaining miRNAs, only a weak experimental association to their putative targets exist.

### Mirna families

MiRNA families are classified based on the mature miRNA sequence similarities and/or structure of the miRNA precursor^34^. There have been evidences that miRNAs within the same family have similar expression patterns and coordinately regulate cell biological processes^35^, ensuring robustness to gene regulatory networks^36^. In the present study, we further performed a global analysis of miRNA families in the two biofluids of HCC patients, in order to identify potential functional correlations among miRNAs. Fifty-eight miRNA families are present in blood with at least two members. The top expressed families are the let-7/98/4458/4500, miR-378/422a/378bcdefhi, miR-17/17-5p/20ab/20b-5p/93/106ab/427/518a-3p/519d, miR-15abc/16/16abc/195/322/424/ 497/1907, miR-181abcd/4262, miR-25/32/92abc/363/363-3p/367 and the miR-320abcd/4429 family. Twenty-four families are entirely represented in blood (Table S3) including the top ranked miR-320abcd/4429 family.

In serum 214 miRNA families are present. The let-7/98/4458/4500, miR-320abcd/4429, miR-378/422a/378bcdefhi and miR-17/17-5p/20ab/20b-5p/93/106ab/427/518a-3p/519d families are the most represented families. Six families are fully represented in serum (Table S3) including the top ranked miR-320abcd/4429 family, as for blood.

**Figure 4:**
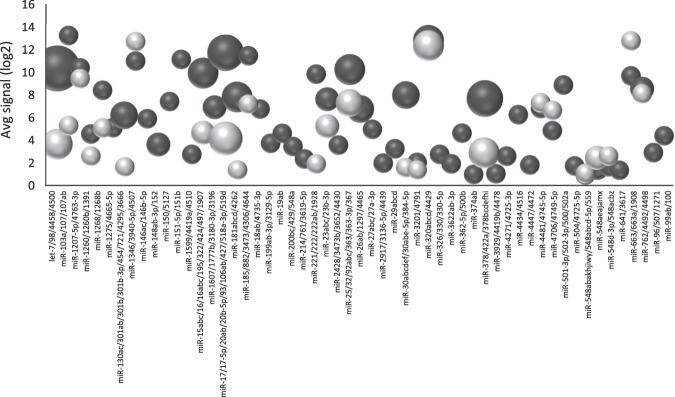
Mirna families in whole blood (black circles) and serum (grey circles). The area of the circle is proportional to the number of the family members expressed. The height correspond to the average log2 signal of the miRNA members within the family.

MiRNA families are differently expressed in the two biofluids. The let-7 family and the miR-378 family are mostly expressed in blood, in which their expression is higher compared to serum (Fig. 4). Furthermore, there are some overlapping families between the two biofluids such as the miR-320 and the miR-548abakhjiwy families (Fig. 4).

**Figure 5:**
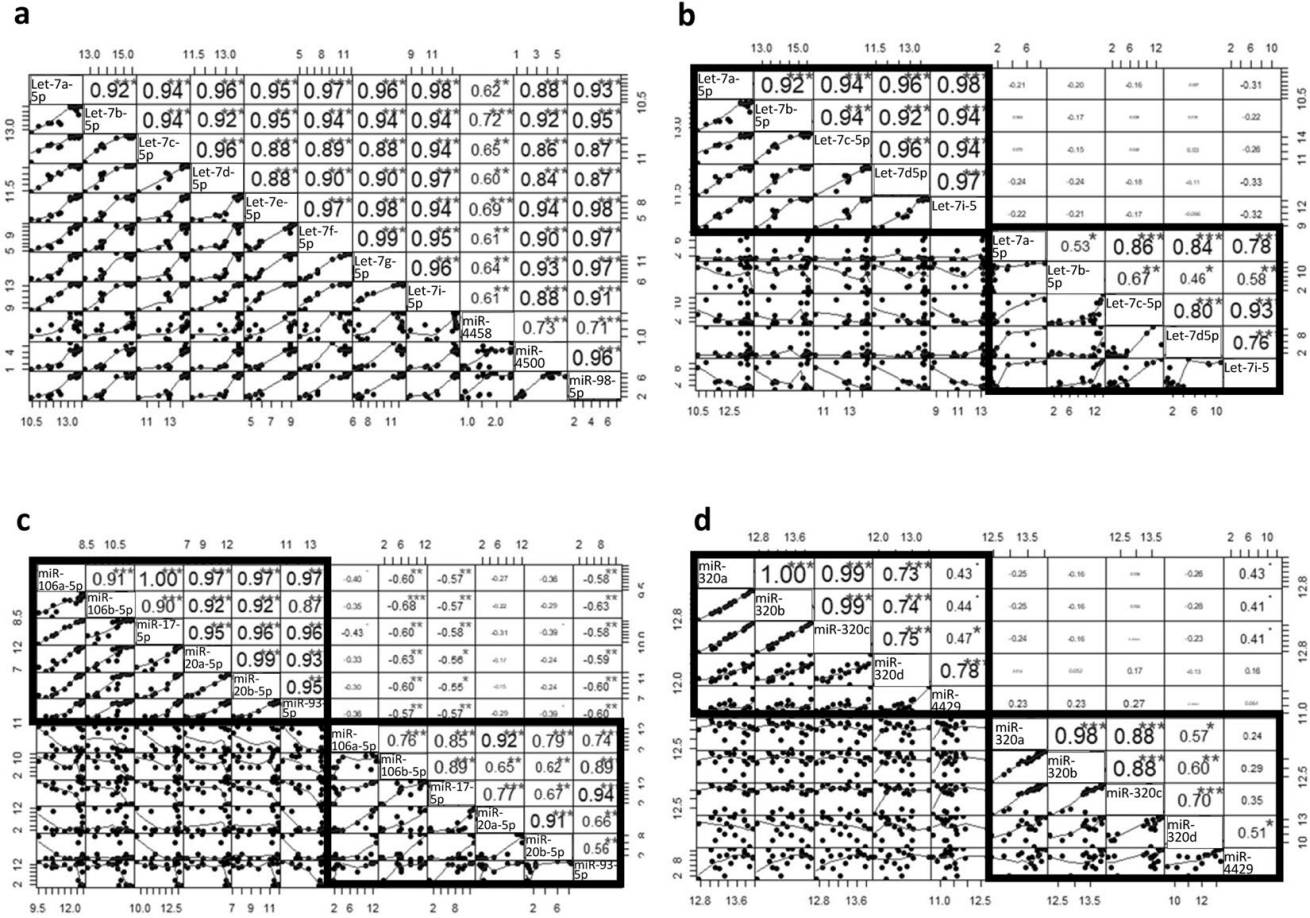
(**a**) Correlation chart of the let7 family members in whole blood. Correlation chart of the let7 family (**b**), miR-17 family (**c**), and miR-320 family members (**d**) in both whole blood and serum. The upper-right side shows the correlation coefficient and p-value for each pairwise correlation. The lower-left side show the distribution. b,c and d show the correlation for the miRNA that are expressed in both biofluids. Intra-blood correlations (black quadrants) and intra-serum correlations (grey quadrants) are reported. Outside the quadrants the pairwise inter-biofluids correlations.

In humans the let-7 family consist of 14 miRNA members, let-7a,b,c,d,e,f,g,i, miR-98, miR-4458 and miR-4500 normally involved in hematopoietic stem progenitor cell homeostasis^37^. In cancers the expression is commonly down-regulated^38^. In HCC evidences of reduced let-7 family members was observed in tissues^39,40^. On the contrary, in serum those miRNA have been shown to be increased^41^. In our blood samples the let-7 family members are highly represented and have a high average expression (avg log2 expr. 10.35; min –max 8.87-15.27 for the let miRNAs, except for miR-4458 (avg log2 1.67; min-max 0.77-2.52) Table S3). The family members correlate in the expression in blood except miR-4458 that has the lowest correlation coefficient (Fig. 5a). The high correlation is also explained by the overlapping among validated mRNA targets (Fig. S7). This observation agrees with the important role of the family in the hematic cells and with the previous finding that reported those miRNA highly expressed in blood^18^. Interestingly, when comparing blood and serum, we observed no correlation in the expression among the blood and serum miRNAs (Fig. 5b), suggesting a possible extra-blood origin of the serum miRNAs. To notice that the overall correlation among the serum miRNA is generally lower than in blood (Fig. 5b).

Another highly correlated family in blood and serum is the miR-17 family. The family members miR-106a-5p, miR-106b-5p, miR-17-5p, miR-20b-5p, miR-93-5p are expressed in the two biofluids. However, while the expression in blood is homogeneous, more variability was observed in serum (Table S3 and Fig. 1, 2). Comparing the two biofluids, we found a negative correlation among serum miR-17-5p, -106b-5p, miR-93-5p and the miRNA family members in blood (Fig. 5c), suggesting two distinctive patterns for the two compartments. MiR-17-5p, -106b-5p, -93-5p have been extensively studied both in HCC tissues^42^ and serum^43,44^, in which they are considered as oncomirs. Thus, their expression in serum could be influenced by the disease rather than blood cells. The high and relatively stable expression of these miRNA suggests a physiological role in blood cell. A high and stable expression of the miR-320 family was observed in blood. Moreover, a high intra-blood and intra-serum exist among the miR-320a, miR-320b and miR-320c members, probably due to the high conservation among the sequences (Fig. 5d). Although the very similar pattern, there is no significant correlation between the two biofluids for the three miRNAs. Only a slightly positive association was observed among serum miR-4429 and the blood miR-320a,b,c.

To the miR-378 family belong miR-378a,b,c,d,e,f,h,I and miR-422 all expressed in blood, while in serum only miR-378a,c,h,I are detectable (Table S3). MiR-378a has been reported to be involved in cell survival, tumor growth and angiogenesis^45^. In liver it inhibits the hepatic stellate cell activation^46^ and works as an onco-suppressor by targeting VEGFR, PDGFRβ and c-Raf^47^. Very few information exist about the other family members, despite their high expression in blood (miR-378c,d,f,i) and serum (miR-378h). MiR-378h has a 47.02 fold higher expression in serum compared to blood (Table S2), however, to our knowledge no information about this miRNA is available in literature. Interestingly miR-378h was previously detected only in liver and testis (Fig. S8). The weakly associated target genes, downloaded from MirTarBase, link this miRNA to FAS signaling pathway, VEGF signaling pathway, Insulin/IGF pathway-protein kinase B signaling cascade, p53 pathway feedback loops 2, Oxidative stress response, Gonadotropin-releasing hormone receptor pathway, Angiogenesis, although no statistically enrichment was obtained (Table S4). To further clarify the function of this miRNA in HCC more extensively studies are needed.

### MiRNA differently expressed in HCC

To further verify the consistency of our data with previously identified miRNA HCC, we searched in StarBase v.3.0 database for differently expressed miRNAs in 370 HCC compared to 50 normal livers. Among the 187 miRNAs expressed in the serum of our patients, we identified 139 being already identified in HCC tissues, however only 95 with a log2(RPM + 0.01) > 0.1 (Table S5). Twenty-five miRNAs were significantly up-regulated in HCC tissues compared to normal liver, while 40 were significantly downregulated (Table S5). With this preliminary study we are not able to define a clinical significance for these findings that should be further validated in independent translational studies.

## Conclusions

In recent years, circulating biomarkers have been considered as a powerful, non-invasive tool for disease detection or cancers follow-up. MiRNAs are eligible biomarker candidates for their stability, easy assessment and role in gene regulation. Although detectable in all biofluids, serum is the most studied source as readily accessible as fresh or frozen material. A portion of cell-free circulating miRNAs released in serum could originate from cancerous cells during tumour progression being promising candidates as cancer biomarkers.

Recently, an increasing interest is paid in the study of the whole blood miRNome^6,48^ originating from monocytes, platelets, mature red blood cells^7^, cells involved in cancer immune-surveillance^8^ and circulating cancer cells. Giving the systemic nature of cancer, the whole blood miRNome could represent an important source of information for biomarker discovery studies. In this work we compared the circulating miRNome from two distinct compartments: Whole blood and serum. Despite the multitude of circulatory miRNAs investigations being present in literature, still fragmented and scanty information exists on HCC. We identified different expression patterns for the two biofluids. Some miRNAs, previously identified as serum biomarkers in HCC are also highly expressed in blood, such as miR-107, miR-92a-3p^20^, miR-17-5p, miR-223^44^. On the other hand, the role and clinical significance of some highly expressed miRNAs in serum are still unexplored in HCC, such as miR-4484, miR-940, miR-933, miR-378h, providing hints for new researches in this field. Despite the major limitation of our study consisting in the relatively low number of patients, we believe that this study might represent a step in the way for future studies enrolling larger cohorts for an independent validation. Our contribution in the characterization of the circulating miRNome in blood and serum from HCC patients is still exploratory and clinical and functional studies are desired to further promote the use of miRNAs as biomarkers in a clinical setting.

## Supplementary information

Supplementary information

Supporting file 1

Supporting file 2

Supporting file 3

## Data Availability

The datasets used during the current study are available from the corresponding author on reasonable request.
