# Supplementary material for: A comparative characterization of the circulating miRNome in whole blood and serum of HCC patients
Source: Sci Rep. 2019 Jun 4;9:8265. doi: 10.1038/s41598-019-44580-x (PMC6547851; doi:10.1038/s41598-019-44580-x)
Supplement: Supplementary file 1 — Supplementary information [file 41598_2019_44580_MOESM1_ESM.pdf]

A comparative characterization of the circulating miRNome in whole blood and serum of HCC patients

Devis Pascut[1*], Helena Krmac[2], Francesca Gilardi[1], Riccardo Patti[3,4], Raffaella Calligaris[2], Lory Saveria Crocè[1,3,4], and Claudio Tiribelli[1]

1. Fondazione Italiana Fegato - ONLUS, Area Science Park, ss14, km163.5 34149, Trieste
2. Scuola Internazionale Superiore di Studi Avanzati - via Bonomea, 265 - 34136 Trieste ITALY
3. Department of Medical Sciences, University of Trieste, Trieste, Italy
4. Clinica Patologie Fegato, Azienda Sanitaria Universitaria Integrata di Trieste (ASUITS), Via Giovanni Sai 7, Trieste, Italy

*__Corresponding Author:__

Dr. Devis Pascut Ph.D.
Liver Research Center
Fondazione Italiana Fegato
Bldg Q AREA Science Park - Basovizza Campus
ss 14 km 163.5 - 34149 Trieste, Italy
phone +39 040 375 7840
fax +39 040 375 7832
Email devis.pascut@fegato.it

SUPPLEMENTARY TABLES pag. 2-22

SUPPLEMENTARY FIGURES pag. 23-29

SUPPLEMENTARY INFORMATION

SUPPLEMENTARY TABLES

Table S1. Demographic and clinical characteristics of the studied populations

	HCC		HCC
**Age (median, 95%LCL-95%UCL)**	68,64.97-71.45	**CLIP**	
**Sex (M/F)**		0	8 (40%)
Male	17 (85%)	1	10 (50%)
Female	3 (15%)	2	2 (10%)
**Cirrhosis yes/no**	20/0	**GRETCH**	
**Aetiology**		Low	12 (60%)
Alcohol metabolic	16 (80%)	Intermediate	8 (40%)
Viral	4 (20%)	**Number of lesions**	
**Child-Pugh**		Single < 2cm	3 (15%)
A	14 (70%)	Single or 3 $\leq$3cm	11 (55%)
B	6 (30%)	Large-single or multi	6 (30%)
**BLCC**		**Alpha fetoprotein**	
0	3 (15%)	<20 ng/mL	15 (75%)
A	12 (60%)	20 - 400 ng/mL	5 (25%)
B	5 (25%)		

Table S2. Differently expressed miRNA list between Blood (B.) and Serum (S.). Gene-Level Fold Change (FC) < -2 or > 2. Gene-Level Anova P-Value < 0.05.

miRNA transcript	B. Bi-weight Avg Sig (log2)	S. Bi-weight Avg Sig(log2)	B. SD	S. SD	FC (linear) (B *vs*. S)	ANOVA p-value	FDR p-value
hsa-miR-15b-5p	13.72	1.23	0.48	2.7	5728.29	6.04E-22	2.96E-20
hsa-let-7a-5p	13.54	1.32	1.04	2.19	4767.91	1.63E-22	8.51E-21
hsa-let-7d-5p	13.64	1.74	0.67	2.93	3824.29	1.32E-18	4.95E-17
hsa-let-7c-5p	14.16	2.42	1.04	3.84	3419.89	1.78E-13	4.30E-12
hsa-miR-26a-5p	12.93	1.34	0.7	4.08	3067.99	5.77E-13	1.34E-11
hsa-let-7i-5p	13.32	2.12	1.6	3.73	2354.86	1.21E-11	2.36E-10
hsa-miR-181a-5p	12.74	1.54	0.83	2.51	2352.35	4.31E-19	1.69E-17
hsa-miR-151a-5p	12.31	1.37	0.36	3.15	1955.05	1.57E-16	4.76E-15
hsa-miR-106a-5p	12.77	2.18	1.12	4.65	1536.61	9.75E-08	1.13E-06
hsa-miR-20b-5p	11.52	1.16	1.75	2.69	1312.17	4.70E-14	1.20E-12
hsa-miR-20a-5p	11.9	1.88	1.98	4.16	1037.89	1.05E-07	1.19E-06
hsa-miR-222-3p	10.78	1.61	0.95	2.9	576.84	4.09E-14	1.06E-12
hsa-miR-126-3p	10.49	1.34	2.69	3.18	566.58	7.22E-08	8.48E-07
hsa-miR-342-3p	10.55	1.42	1	2.46	561.89	8.77E-18	3.02E-16
hsa-miR-150-5p	10.63	1.63	1.53	2.45	511.61	2.64E-16	7.91E-15
hsa-miR-15a-5p	10.22	1.28	1.91	0.6	490.56	2.25E-20	9.53E-19
hsa-miR-532-5p	9.99	1.3	0.85	0.74	413.92	1.58E-30	3.08E-28
hsa-miR-320e	10.21	1.67	0.46	3.1	371.79	2.38E-12	5.12E-11
hsa-miR-23b-3p	10.27	1.87	0.44	3.78	337.21	6.58E-09	8.77E-08
hsa-miR-30d-5p	10.36	2.01	0.8	1.72	324.73	2.76E-21	1.26E-19
hsa-miR-140-3p	12.77	4.45	0.62	4.48	318.2	3.61E-09	5.03E-08
hsa-miR-103a-3p	13.4	5.08	0.27	4.53	318.11	8.31E-10	1.25E-08
hsa-miR-502-3p	9.44	1.2	0.74	0.98	301.47	5.01E-29	6.88E-27
hsa-miR-4306	9.64	1.45	0.72	1.83	292.26	1.40E-20	6.13E-19
hsa-miR-378a-3p	10.2	2.07	0.56	3.12	280.28	3.74E-11	6.94E-10
hsa-miR-584-5p	9.67	1.63	0.71	1.55	261.97	1.86E-21	8.76E-20
hsa-miR-130b-3p	10.11	2.17	0.47	3.24	245.54	9.80E-11	1.71E-09
hsa-miR-574-3p	9.89	2.1	1	2.11	220.19	4.75E-17	1.51E-15
hsa-miR-130a-3p	9.13	1.39	1.23	2.29	213.88	7.31E-15	2.00E-13
hsa-miR-378c	9.21	1.63	0.64	1.41	191.89	1.47E-22	7.77E-21
hsa-miR-107	13.17	5.6	0.33	4.63	190.07	7.67E-09	1.01E-07
hsa-miR-4492	10.39	2.85	0.77	4.3	185.57	7.24E-07	7.41E-06
hsa-miR-378i	8.74	1.24	0.76	1.34	181.1	1.47E-22	7.77E-21
hsa-miR-145-5p	8.56	1.16	1.05	2.35	168.35	6.43E-15	1.78E-13
hsa-miR-423-5p	11.38	4.13	0.84	3.49	152.37	2.29E-10	3.77E-09
hsa-miR-223-3p	8.78	1.53	1.2	2.51	152.21	4.51E-12	9.41E-11
hsa-miR-378f	8.5	1.31	0.82	1.24	147.01	2.14E-22	1.08E-20
hsa-miR-155-5p	8.23	1.29	0.6	4.56	122.75	0.0001	0.0007
hsa-miR-18a-5p	8.09	1.29	2.08	1.36	111.79	1.44E-12	3.19E-11
hsa-miR-550a-3p	7.79	1.14	1.16	1	100.64	1.83E-22	9.40E-21
hsa-miR-151a-3p	8.67	2.08	0.87	2.89	96.4	1.45E-10	2.45E-09
hsa-miR-221-3p	8.9	2.45	0.96	4.35	87.13	0.0004	0.0021

hsa-miR-361-5p	7.96	1.65	0.79	1.89	79.39	1.01E-15	2.93E-14
hsa-miR-125b-5p	7.58	1.28	1.52	0.44	79.25	2.29E-21	1.06E-19
hsa-miR-503-5p	7.58	1.45	1.12	0.41	70.25	3.12E-24	1.98E-22
hsa-miR-17-5p	12.92	6.86	1.09	4.91	66.92	6.00E-06	5.19E-05
hsa-miR-1307-3p	7.93	1.91	0.49	3.39	64.93	6.99E-07	7.18E-06
hsa-miR-181b-5p	7.46	1.45	0.47	0.7	64.42	3.30E-30	5.87E-28
hsa-miR-197-3p	7.35	1.57	0.97	1.11	55.15	5.34E-20	2.17E-18
hsa-miR-4530	12.36	6.69	0.7	4	50.99	2.44E-07	2.67E-06
hsa-miR-3135b	7.89	2.56	0.92	3.7	40.22	7.85E-05	0.0005
hsa-miR-1268b	8.31	2.99	0.42	4.37	39.95	0.0054	0.0159
hsa-miR-106b-5p	10.87	5.56	0.93	4.05	39.78	2.17E-06	2.03E-05
hsa-miR-30a-5p	6.32	1.32	0.73	0.92	32.13	1.99E-21	9.27E-20
hsa-miR-92b-3p	10.3	5.32	0.73	3.2	31.56	1.04E-08	1.36E-07
hsa-miR-4690-5p	7.86	2.89	0.87	2.96	31.38	6.98E-07	7.18E-06
hsa-miR-574-5p	6.31	1.37	1.57	1.59	30.56	1.80E-09	2.61E-08
hsa-miR-93-5p	14.06	9.13	1.19	5.13	30.43	7.93E-06	6.59E-05
hsa-miR-1225-5p	6.86	1.94	0.79	3.92	30.21	0.0057	0.0165
hsa-miR-191-5p	14.06	9.33	0.3	5.27	26.49	4.56E-07	4.84E-06
hsa-miR-3162-5p	6.17	1.46	0.77	2.03	26.19	6.55E-10	1.01E-08
hsa-miR-371b-5p	6.33	1.71	1.28	2.73	24.49	3.34E-05	0.0002
hsa-miR-1910-5p	6.75	2.23	0.45	2.74	22.94	2.11E-06	1.98E-05
hsa-miR-25-3p	13.26	8.77	0.3	5.12	22.41	2.83E-06	2.60E-05
hsa-miR-29a-3p	5.62	1.2	1.19	1.86	21.39	5.07E-09	6.86E-08
hsa-miR-664a-5p	5.53	1.16	1.24	0.33	20.75	5.77E-17	1.82E-15
hsa-let-7b-5p	15.4	11.11	0.95	4.64	19.55	8.71E-07	8.72E-06
hsa-miR-193a-5p	6.44	2.17	0.74	3.15	19.28	0.0007	0.0031
hsa-miR-885-3p	5.82	1.6	1.08	2.58	18.64	4.10E-05	0.0003
hsa-miR-491-5p	5.47	1.29	0.9	0.45	18.12	4.54E-21	2.05E-19
hsa-miR-1224-5p	5.58	1.49	0.59	2.69	16.99	1.27E-05	0.0001
hsa-miR-3141	7.8	3.86	0.64	4.18	15.34	0.0084	0.0226
hsa-miR-1207-5p	9.6	5.76	0.63	4.02	14.33	0.0002	0.0011
hsa-miR-4505	10.99	7.24	0.57	3.88	13.49	6.97E-05	0.0005
hsa-miR-4800-3p	5.12	1.38	0.72	1.28	13.33	5.54E-12	1.13E-10
hsa-miR-27a-3p	5.12	1.4	0.99	2.01	13.18	2.19E-06	2.05E-05
hsa-miR-4539	5.82	2.17	0.9	2.11	12.51	3.15E-06	2.87E-05
hsa-miR-409-3p	5.21	1.68	1.38	1.31	11.5	1.01E-07	1.17E-06
hsa-miR-1909-5p	4.73	1.45	0.66	0.68	9.72	1.06E-16	3.24E-15
hsa-miR-4443	10.31	7.09	0.76	4.14	9.28	0.0008	0.0035
hsa-miR-629-3p	4.56	1.42	1.7	0.97	8.81	6.46E-08	7.66E-07
has-mir-451-3p	12.21	9.14	0.74	4.7	8.38	0.0001	0.0008
hsa-miR-885-5p	4.62	1.55	0.91	1.17	8.37	1.96E-09	2.82E-08
hsa-miR-4741	9.27	6.25	0.74	4.93	8.11	0.0182	0.0411
hsa-miR-1587	8.96	5.98	0.78	3.56	7.92	0.0011	0.0046
hsa-miR-1260b	6.26	3.29	0.98	2.12	7.82	2.20E-06	2.05E-05
hsa-miR-4708-5p	4.17	1.27	0.83	0.36	7.49	5.01E-16	1.47E-14
hsa-miR-3679-5p	4.57	1.77	0.8	1.42	6.98	1.80E-07	1.99E-06
hsa-miR-4514	3.66	1.02	0.93	0.29	6.23	1.30E-14	3.50E-13
hsa-miR-4433a-3p	8.09	5.45	0.56	4.03	6.22	0.0163	0.0377

hsa-miR-4286	4.26	1.64	1.13	0.43	6.17	8.53E-12	1.71E-10
hsa-miR-425-5p	12.65	10.13	0.8	5.19	5.77	8.09E-06	6.72E-05
hsa-miR-486-3p	3.5	1.12	1.71	1.16	5.22	1.73E-05	0.0001
hsa-miR-16-5p	13.91	11.57	0.55	5.68	5.08	4.26E-05	0.0003
hsa-miR-4429	11.31	9.01	0.44	3.39	4.91	0.0003	0.0018
hsa-miR-4454	9.16	6.9	2.04	4.56	4.79	0.0011	0.0046
hsa-miR-4430	3.56	1.57	0.59	1.41	3.99	0.0001	0.0008
hsa-miR-3646	3.59	1.7	0.64	1.5	3.72	0.0016	0.0061
hsa-miR-143-3p	2.84	1.09	1.41	0.61	3.37	2.37E-05	0.0002
hsa-miR-3935	3.15	1.54	0.69	0.73	3.04	1.16E-06	1.13E-05
hsa-miR-486-5p	15.39	13.93	0.29	2.63	2.75	0.0009	0.0039
hsa-miR-4481	3.22	1.76	0.68	1.08	2.75	0.0014	0.0054
hsa-miR-1255a	2.59	1.21	1.32	0.47	2.6	0.0002	0.001
hsa-miR-92a-3p	14.84	13.59	0.18	5.15	2.38	0.0003	0.0015
hsa-miR-1915-5p	2.57	1.41	0.39	0.39	2.24	1.14E-11	2.24E-10
hsa-miR-4725-5p	2.26	3.27	0.69	3.29	-2.02	0.0036	0.0115
hsa-miR-3157-3p	2.1	3.24	0.62	2.56	-2.21	0.005	0.0151
hsa-miR-2861	12.57	13.75	0.69	0.37	-2.27	1.57E-07	1.74E-06
hsa-miR-23a-3p	11.21	12.5	0.86	5.57	-2.44	0.0084	0.0225
hsa-miR-4507	11.15	12.54	0.77	1.02	-2.63	0.0007	0.0033
hsa-miR-4466	12.39	13.96	0.66	0.28	-2.97	3.50E-12	7.36E-11
hsa-miR-638	12.44	14.03	0.68	0.25	-3.01	4.81E-12	9.90E-11
hsa-miR-3656	11.91	13.53	0.68	0.62	-3.09	5.02E-09	6.80E-08
hsa-miR-548ac	1.9	3.57	0.74	2.7	-3.19	0.0001	0.0008
hsa-miR-3196	11.68	13.51	0.63	0.99	-3.55	1.50E-06	1.43E-05
hsa-miR-4763-3p	11.24	13.25	0.76	0.66	-4.05	2.37E-10	3.87E-09
hsa-miR-4310	2.45	4.48	0.46	3.08	-4.08	0.0004	0.0021
hsa-miR-762	11.52	13.55	0.65	0.64	-4.09	1.82E-12	3.98E-11
hsa-miR-4463	10.11	12.24	0.62	2.69	-4.36	0.0265	0.055
hsa-miR-3940-5p	10.88	13.01	0.51	1.32	-4.39	1.06E-06	1.04E-05
hsa-miR-4488	11.41	13.59	0.52	0.69	-4.53	2.74E-12	5.85E-11
hsa-miR-2277-5p	3.7	5.89	0.89	3.12	-4.55	0.0016	0.0061
hsa-miR-1908-5p	10.96	13.14	0.41	0.97	-4.56	2.31E-10	3.79E-09
hsa-miR-4516	11.49	13.94	0.86	0.44	-5.47	7.79E-14	1.97E-12
hsa-miR-548ae-3p	1.47	3.95	0.5	2.18	-5.59	6.80E-06	5.76E-05
hsa-miR-4281	9.91	12.45	0.59	2.87	-5.8	0.0225	0.0484
hsa-miR-4745-5p	10.45	13.11	0.64	3.11	-6.3	0.0284	0.0581
hsa-miR-4497	11.15	13.96	0.74	0.37	-7.05	3.22E-18	1.15E-16
hsa-miR-4274	2.49	5.39	0.63	2.12	-7.47	7.05E-07	7.22E-06
hsa-miR-1469	10.2	13.58	0.62	0.43	-10.39	3.93E-22	1.95E-20
hsa-miR-4674	8.09	11.71	0.76	3.73	-12.32	0.0031	0.0104
hsa-miR-4707-5p	9.15	12.93	0.73	0.73	-13.69	7.24E-19	2.76E-17
hsa-let-7f-1-3p	2.14	6.12	0.65	3.39	-15.82	4.41E-06	3.95E-05
hsa-miR-548a-3p	2.41	6.57	0.82	3.22	-17.97	1.21E-06	1.18E-05
hsa-miR-933	3.63	7.87	0.67	3.54	-18.83	6.34E-06	5.45E-05
hsa-miR-663a	8.3	12.64	0.51	2.92	-20.14	3.93E-05	0.0003
hsa-miR-3921	2.12	6.65	0.83	2.85	-22.99	2.05E-07	2.26E-06
hsa-miR-3619-5p	3.46	8.11	0.43	3.03	-25.11	3.21E-07	3.49E-06

hsa-miR-3613-5p	2.27	7.06	2.34	3.59	-27.65	0.0003	0.0015
hsa-miR-191-3p	3.24	8.06	0.74	4.12	-28.2	9.70E-06	7.92E-05
hsa-miR-4529-3p	2.66	7.96	0.77	3.25	-39.29	1.01E-07	1.16E-06
hsa-miR-378h	2.89	8.45	0.62	2.64	-47.02	7.76E-12	1.56E-10
hsa-miR-1280	6.61	12.63	1.02	1.73	-64.82	3.10E-15	8.73E-14
hsa-miR-4706	4.37	10.41	0.58	2.35	-66.08	9.13E-13	2.08E-11
hsa-miR-3613-3p	7.72	13.82	1.37	0.81	-68.61	1.82E-20	7.78E-19
hsa-miR-4668-5p	7.61	13.95	1.74	0.59	-80.91	4.77E-19	1.86E-17
hsa-miR-1184	3.43	9.8	0.97	3.5	-82.89	1.39E-08	1.81E-07
hsa-miR-1228-3p	3.49	10.09	0.82	2.69	-97.01	5.49E-12	1.13E-10
hsa-miR-4532	6.83	13.9	0.76	0.38	-134.24	6.31E-33	1.69E-30
hsa-miR-1281	6.66	13.79	1.12	0.49	-139.36	6.96E-27	6.33E-25
hsa-miR-3178	6.33	13.45	0.62	0.86	-139.75	7.48E-29	9.50E-27
hsa-miR-940	4.12	11.6	0.85	1.46	-178.7	2.83E-22	1.41E-20
hsa-miR-455-3p	5.34	13.12	1.25	1.48	-219.11	2.39E-20	1.00E-18
hsa-miR-122-5p	4.93	13.38	1.56	4.7	-348.64	2.16E-06	2.02E-05
hsa-miR-1825	4.88	13.39	0.92	1.13	-365.17	7.82E-26	5.73E-24
hsa-miR-4487	3.99	12.75	0.81	0.88	-432.58	4.66E-30	7.51E-28
hsa-miR-4484	5.04	14.34	1.01	0.19	-633.09	1.58E-34	4.47E-32

Table S3: miRNA families expressed in whole blood and serum

FAMILIES IN WHOLE BLOOD				
**Family**	**let-7/98/4458/4500**			
**11/11**	**Mean avg log2 signal**	**Median avg log2 signal**	**Min**	**Max**
hsa-let-7a-5p	12.898486	13.450455	10.45187	13.94199
hsa-let-7b-5p	14.7157475	15.279165	12.70712	15.67529
hsa-let-7c-5p	13.575469	14.077915	10.35575	14.50035
hsa-let-7d-5p	13.221659	13.5897	11.4002	13.78844
hsa-let-7e-5p	7.6171803	8.753424	4.453341	9.432898
hsa-let-7f-5p	9.4127488	10.86407	4.983291	11.83872
hsa-let-7g-5p	10.4320745	12.015415	6.057606	12.62968
hsa-let-7i-5p	12.25869645	13.21787	8.624542	13.49819
hsa-miR-4458	1.672109205	1.6576885	0.7624265	2.523499
hsa-miR-4500	3.310374735	4.1290715	0.872941	5.704439
hsa-miR-98-5p	5.04120085	6.2376825	1.316216	7.282755
**Family**	**miR-103a/107/107ab**			
**2/2**	**Mean avg log2 signal**	**Median avg log2 signal**	**Min**	**Max**
hsa-miR-103a-3p	13.3174205	13.338685	12.67991	13.74401
hsa-miR-107	13.035596	13.08912	12.14367	13.44128
**Family**	**miR-1207-5p/4763-3p**			
**2/2**	**Mean avg log2 signal**	**Median avg log2 signal**	**Min**	**Max**
hsa-miR-1207-5p	9.6653736	9.569501	8.532763	10.99559
hsa-miR-4763-3p	11.1581676	11.202305	9.837146	12.48545
**Family**	**miR-130ac/301ab/301b/301b-3p/454/721/4295/3666**			
**4/4**	**Mean avg log2 signal**	**Median avg log2 signal**	**Min**	**Max**
hsa-miR-130a-3p	8.88228835	9.341063	6.95453	10.32921
hsa-miR-130b-3p	9.9758087	10.08863	9.142858	10.70487
hsa-miR-301a-3p	1.1125341	1.108107	0.6327404	2.363882
hsa-miR-454-3p	4.73473102	6.120035	0.6522533	8.081027
**Family**	**miR-1260/1260b/1391**			
**2/2**	**Mean avg log2 signal**	**Median avg log2 signal**	**Min**	**Max**
hsa-miR-1260a	3.15088185	2.9304065	1.560665	5.077653
hsa-miR-1260b	6.3962241	6.0939625	5.107658	8.03319
**Family**	**miR-1268/1268b**			
**2/2**	**Mean avg log2 signal**	**Median avg log2 signal**	**Min**	**Max**
hsa-miR-1268a	8.6119876	8.663597	7.666307	9.54143
hsa-miR-1268b	8.2284553	8.305124	7.381428	9.191389
**Family**	**miR-1275/4665-5p**			
**2/2**	**Mean avg log2 signal**	**Median avg log2 signal**	**Min**	**Max**
hsa-miR-1275	4.612554	4.5526735	2.376538	6.309568
hsa-miR-4665-5p	5.45970655	5.4214115	4.409567	6.900012

Family	miR-1346/3940-5p/4507			
2/2	Mean avg log2 signal	Median avg log2 signal	Min	Max
hsa-miR-3940-5p	10.807938	10.83619	9.58687	12.02106
hsa-miR-4507	10.9915856	11.192245	9.587625	12.46904
**Family**	**miR-146ac/146b-5p**			
**2/2**	**Mean avg log2 signal**	**Median avg log2 signal**	**Min**	**Max**
hsa-miR-146a-5p	6.9529938	7.299675	3.47011	8.793442
hsa-miR-146b-5p	4.8173639	6.0506235	1.112573	7.663585
**Family**	**miR-148ab-3p/152**			
**3/3**	**Mean avg log2 signal**	**Median avg log2 signal**	**Min**	**Max**
hsa-miR-148a-3p	2.7609717	2.9900775	1.032141	4.280493
hsa-miR-148b-3p	5.6649989	5.7930435	4.280577	6.914677
hsa-miR-152-3p	3.216250375	3.334524	0.8434375	5.672692
**Family**	**miR-15abc/16/16abc/195/322/424/497/1907**			
**5/6**	**Mean avg log2 signal**	**Median avg log2 signal**	**Min**	**Max**
hsa-miR-15a-5p	9.01949075	10.004165	5.429198	11.17664
hsa-miR-15b-5p	13.5908935	13.724665	11.87652	14.10804
hsa-miR-16-5p	13.846191	14.00892	12.8509	14.96003
hsa-miR-195-5p	3.84092482	4.361587	0.6343706	6.061136
hsa-miR-497-5p	1.34810348	1.3513955	0.7612849	2.231621
**Family**	**miR-151-5p/151b**			
**2/2**	**Mean avg log2 signal**	**Median avg log2 signal**	**Min**	**Max**
hsa-miR-151a-5p	12.2124315	12.2629	11.44741	12.94877
hsa-miR-151b	9.713841	9.957894	8.38445	10.70801
**Family**	**miR-1599/4419a/4510**			
**2/2**	**Mean avg log2 signal**	**Median avg log2 signal**	**Min**	**Max**
hsa-miR-4419a	1.379541575	1.3695045	0.7510089	2.73024
hsa-miR-4510	3.94564115	4.295832	1.866084	5.831294
**Family**	**miR-1607/1777b/3180-3p/3196**			
**3/3**	**Mean avg log2 signal**	**Median avg log2 signal**	**Min**	**Max**
hsa-miR-3180	3.89449805	4.1065495	1.906312	5.316168
hsa-miR-3180-3p	4.25329525	4.36974	2.051952	5.709913
hsa-miR-3196	11.5846967	11.58531	9.775994	12.46537
**Family**	**miR-17/17-5p/20ab/20b-5p/93/106ab/427/518a-3p/519d**			
**6/7**	**Mean avg log2 signal**	**Median avg log2 signal**	**Min**	**Max**
hsa-miR-106a-5p	12.0548603	12.64541	9.456276	13.01545
hsa-miR-106b-5p	10.5454851	10.768135	8.458914	11.69474
hsa-miR-17-5p	12.2766962	12.817355	9.643184	13.22032
hsa-miR-20a-5p	10.6389435	11.702195	6.606642	12.32075
hsa-miR-20b-5p	10.41014615	11.3175	7.120128	11.92689
hsa-miR-93-5p	13.3162845	13.935965	10.59467	14.51626

Family	miR-181abcd/4262			
4/5	Mean avg log2 signal	Median avg log2 signal	Min	Max
hsa-miR-181a-5p	12.237075	12.60924	10.40391	13.03827
hsa-miR-181b-5p	7.4308859	7.452602	6.694617	8.331334
hsa-miR-181c-5p	7.05770095	7.229053	4.205859	8.733038
hsa-miR-181d-5p	2.479373195	2.66759	0.7371904	3.693894
Family	miR-185/882/3473/4306/4644			
2/2	Mean avg log2 signal	Median avg log2 signal	Min	Max
hsa-miR-185-5p	13.6131415	13.612415	12.94532	14.43655
hsa-miR-4306	9.5851533	9.625123	8.573886	11.08395
Family	miR-18ab/4735-3p			
2/3	Mean avg log2 signal	Median avg log2 signal	Min	Max
hsa-miR-18a-5p	7.25743645	7.9395465	2.650928	9.418316
hsa-miR-18b-5p	4.86504455	5.6011915	1.255617	7.00293
Family	miR-199ab-3p/3129-5p			
3/3	Mean avg log2 signal	Median avg log2 signal	Min	Max
hsa-miR-199a-3p	5.039588145	6.8201855	0.7457011	8.302681
hsa-miR-199b-3p	5.01880496	6.840994	0.7598579	8.365027
hsa-miR-3129-5p	1.082927915	1.0715895	0.6388933	1.973848
Family	miR-19ab			
2/2	Mean avg log2 signal	Median avg log2 signal	Min	Max
hsa-miR-19a-3p	1.761639125	1.63291	0.822248	4.084675
hsa-miR-19b-3p	8.4384416	8.3023215	6.276962	12.93111
Family	miR-200bc/429/548a			
2/3	Mean avg log2 signal	Median avg log2 signal	Min	Max
hsa-miR-200b-3p	0.96560022	0.90483135	0.5848499	1.634786
hsa-miR-200c-3p	6.0023042	5.939317	5.236562	7.358823
Family	miR-214/761/3619-5p			
2/3	Mean avg log2 signal	Median avg log2 signal	Min	Max
hsa-miR-214-3p	1.474617935	1.26854	0.6382844	3.714994
hsa-miR-3619-5p	3.3383822	3.381099	2.603078	4.23382
Family	miR-221/222/222ab/1928			
2/2	Mean avg log2 signal	Median avg log2 signal	Min	Max
hsa-miR-221-3p	8.8113346	8.791164	6.99317	10.19602
hsa-miR-222-3p	10.35618355	10.697535	8.079048	11.37158
Family	miR-23abc/23b-3p			
3/3	Mean avg log2 signal	Median avg log2 signal	Min	Max
hsa-miR-23a-3p	11.4533595	11.082025	10.46935	12.79593
hsa-miR-23b-3p	10.26227085	10.177485	9.5832	11.21023
hsa-miR-23c	1.9310684	1.8444625	1.299981	3.425299
Family	miR-2428/3473b/3652/4430			

2/2	Mean avg log2 signal	Median avg log2 signal	Min	Max
hsa-miR-3652	3.27126695	3.4849615	1.600883	4.268707
hsa-miR-4430	3.4661872	3.665273	2.164359	4.308377
**Family**	**miR-25/32/92abc/363/363-3p/367**			
**5/6**	**Mean avg log2 signal**	**Median avg log2 signal**	**Min**	**Max**
hsa-miR-25-3p	13.2250475	13.17894	12.44407	13.88603
hsa-miR-32-5p	1.236289285	1.2425095	0.681765	1.991363
hsa-miR-363-3p	9.88422065	10.52155	6.646354	11.17271
hsa-miR-92a-3p	14.8355845	14.814775	14.23974	15.15251
hsa-miR-92b-3p	10.4484363	10.14703	9.273262	12.10224
**Family**	**miR-26ab/1297/4465**			
**3/4**	**Mean avg log2 signal**	**Median avg log2 signal**	**Min**	**Max**
hsa-miR-26a-5p	12.7091785	12.843085	10.85699	13.57324
hsa-miR-26b-5p	5.5979821	7.327936	1.003913	8.665193
hsa-miR-4465	1.205791975	1.1331895	0.6287912	1.881693
**Family**	**miR-27abc/27a-3p**			
**2/2**	**Mean avg log2 signal**	**Median avg log2 signal**	**Min**	**Max**
hsa-miR-27a-3p	5.08080575	5.0539825	2.995015	7.040871
hsa-miR-27b-3p	4.46086415	5.230926	1.20245	6.585092
**Family**	**miR-2917/3136-5p/4439**			
**2/2**	**Mean avg log2 signal**	**Median avg log2 signal**	**Min**	**Max**
hsa-miR-3136-5p	2.67716665	3.0741135	0.761285	4.617513
hsa-miR-4439	1.09546422	1.0443235	0.6915876	1.93079
**Family**	**miR-29abcd**			
**3/3**	**Mean avg log2 signal**	**Median avg log2 signal**	**Min**	**Max**
hsa-miR-29a-3p	5.3892634	5.494406	1.787211	6.962849
hsa-miR-29b-3p	1.2963238	1.2037475	0.6151571	2.535277
hsa-miR-29c-3p	1.3877182	1.249247	0.7324927	2.721197
**Family**	**miR-30abcdef/30abe-5p/384-5p**			
**5/5**	**Mean avg log2 signal**	**Median avg log2 signal**	**Min**	**Max**
hsa-miR-30a-5p	6.27376395	6.260433	4.451009	7.526642
hsa-miR-30b-5p	7.7415504	6.86455	5.085124	12.34427
hsa-miR-30c-5p	10.64205735	9.440242	8.276537	13.50391
hsa-miR-30d-5p	10.3848615	10.37724	9.167439	12.38509
hsa-miR-30e-5p	4.18209515	4.4859385	1.504991	5.907619
**Family**	**miR-320abcd/4429**			
**5/5**	**Mean avg log2 signal**	**Median avg log2 signal**	**Min**	**Max**
hsa-miR-320a	13.4507005	13.46148	12.75272	13.9659
hsa-miR-320b	13.4159215	13.421025	12.76114	13.95109
hsa-miR-320c	13.3393435	13.382045	12.73396	13.8791
hsa-miR-320d	12.5435665	12.57789	11.87254	13.4777

hsa-miR-4429	11.385783	11.33137	10.74698	12.71708
**Family**	**miR-3201/4791**			
**2/2**	**Mean avg log2 signal**	**Median avg log2 signal**	**Min**	**Max**
hsa-miR-3201	2.49592025	2.176582	0.913062	6.261142
hsa-miR-4791	1.218232355	1.2117025	0.7351692	2.091576
**Family**	**miR-326/330/330-5p**			
**2/2**	**Mean avg log2 signal**	**Median avg log2 signal**	**Min**	**Max**
hsa-miR-326	3.1760363	2.925112	1.2592	5.720776
hsa-miR-330-5p	2.8148072	3.067998	1.354023	4.336797
**Family**	**miR-3622ab-3p**			
**2/2**	**Mean avg log2 signal**	**Median avg log2 signal**	**Min**	**Max**
hsa-miR-3622a-3p	2.17124619	2.15118	0.7331078	3.26551
hsa-miR-3622b-3p	1.95652625	1.776769	1.159483	3.115204
**Family**	**miR-362-5p/500b**			
**2/2**	**Mean avg log2 signal**	**Median avg log2 signal**	**Min**	**Max**
hsa-miR-362-5p	8.132611	8.067062	7.072094	10.22527
hsa-miR-500b-5p	1.497982825	1.426694	0.615822	3.054981
**Family**	**miR-374ab**			
**2/2**	**Mean avg log2 signal**	**Median avg log2 signal**	**Min**	**Max**
hsa-miR-374a-5p	1.01250771	1.0355985	0.6720278	1.329449
hsa-miR-374b-5p	1.068699655	1.014693	0.6220243	1.987553
**Family**	**miR-378/422a/378bcdefhi**			
**9/9**	**Mean avg log2 signal**	**Median avg log2 signal**	**Min**	**Max**
hsa-miR-378a-3p	9.95457555	10.10124	8.56129	10.63236
hsa-miR-378b	1.84170509	1.853494	0.9340728	2.71809
hsa-miR-378c	8.9287751	9.1786515	7.399598	9.682161
hsa-miR-378d	6.6496475	6.978897	4.141099	7.597953
hsa-miR-378e	3.9771106	4.7374465	1.116264	5.211974
hsa-miR-378f	8.0679857	8.438324	5.702308	8.763234
hsa-miR-378h	2.8103731	2.888118	1.462944	4.287439
hsa-miR-378i	8.22607305	8.610106	6.165626	8.884276
hsa-miR-422a	7.7407268	7.798176	5.75587	8.67894
**Family**	**miR-3929/4419b/4478**			
**2/3**	**Mean avg log2 signal**	**Median avg log2 signal**	**Min**	**Max**
hsa-miR-4419b	1.09843614	1.0708115	0.6315964	1.611429
hsa-miR-4478	1.31105597	1.212673	0.8649595	2.118065
**Family**	**miR-4271/4725-3p**			
**2/2**	**Mean avg log2 signal**	**Median avg log2 signal**	**Min**	**Max**
hsa-miR-4271	2.67177386	2.645739	0.9501572	4.128875
hsa-miR-4725-3p	2.40025962	2.598544	0.9324124	3.375923
**Family**	**miR-4434/4516**			

2/2	Mean avg log2 signal	Median avg log2 signal	Min	Max
hsa-miR-4434	0.96422582	0.9653833	0.5781102	1.576081
hsa-miR-4516	11.5023617	11.411965	9.966064	13.01202
**Family**	**miR-4447/4472**			
**2/2**	**Mean avg log2 signal**	**Median avg log2 signal**	**Min**	**Max**
hsa-miR-4447	2.17316745	2.2142745	1.091441	3.353612
hsa-miR-4472	1.514443605	1.37873	0.7793875	2.39555
**Family**	**miR-4481/4745-5p**			
**2/2**	**Mean avg log2 signal**	**Median avg log2 signal**	**Min**	**Max**
hsa-miR-4481	2.9714048	3.158759	1.334301	3.935843
hsa-miR-4745-5p	10.2985313	10.349565	8.739562	11.55694
**Family**	**miR-4706/4749-5p**			
**2/2**	**Mean avg log2 signal**	**Median avg log2 signal**	**Min**	**Max**
hsa-miR-4706	4.23448315	4.3281415	2.331694	5.07913
hsa-miR-4749-5p	5.0742068	5.1827215	4.45028	5.956599
**Family**	**miR-501-3p/502-3p/500/502a**			
**2/2**	**Mean avg log2 signal**	**Median avg log2 signal**	**Min**	**Max**
hsa-miR-501-3p	8.79339705	8.687498	8.0463	10.32675
hsa-miR-502-3p	9.54387435	9.5669825	8.52847	11.27128
**Family**	**miR-504/4725-5p**			
**2/2**	**Mean avg log2 signal**	**Median avg log2 signal**	**Min**	**Max**
hsa-miR-4725-5p	2.16070075	2.1607765	1.011277	3.509783
hsa-miR-504-5p	1.61891665	1.4653605	0.8359687	3.497378
**Family**	**miR-548abakhjiwy/548abcd-5p/559**			
**2/12**	**Mean avg log2 signal**	**Median avg log2 signal**	**Min**	**Max**
hsa-miR-548ab	1.01595683	1.0043047	0.6218158	1.477092
hsa-miR-548i	1.025059185	0.9844798	0.7193273	1.631554
**Family**	**miR-548aeajamx**			
**3/4**	**Mean avg log2 signal**	**Median avg log2 signal**	**Min**	**Max**
hsa-miR-548ae-3p	1.508722405	1.416429	0.8639881	2.491318
hsa-miR-548aj-3p	1.517676365	1.359319	0.8354787	3.048688
hsa-miR-548x-3p	1.55738969	1.4719475	0.8407043	2.83375
**Family**	**miR-548d-3p/548acbz**			
**2/4**	**Mean avg log2 signal**	**Median avg log2 signal**	**Min**	**Max**
hsa-miR-548ac	1.957020985	1.884735	0.8729837	4.342703
hsa-miR-548z	1.34895855	1.1588105	0.772961	2.974461
**Family**	**miR-641/3617**			
**2/2**	**Mean avg log2 signal**	**Median avg log2 signal**	**Min**	**Max**
hsa-miR-3617-5p	1.233971425	1.197068	0.5541543	1.820657
hsa-miR-641	2.10549725	1.866368	1.145179	5.677279

Family	miR-663/663a/1908			
2/2	Mean avg log2 signal	Median avg log2 signal	Min	Max
hsa-miR-1908-5p	10.8654924	10.93312	9.716728	11.54811
hsa-miR-663a	8.15983015	8.2722395	6.89099	9.13215
**Family**	**miR-762/4492/4498**			
**3/3**	**Mean avg log2 signal**	**Median avg log2 signal**	**Min**	**Max**
hsa-miR-4492	10.3242048	10.36615	8.949589	11.95001
hsa-miR-4498	3.19267263	3.340617	0.9765656	4.137019
hsa-miR-762	11.3565026	11.459765	9.842562	12.35717
**Family**	**miR-96/507/1271**			
**2/2**	**Mean avg log2 signal**	**Median avg log2 signal**	**Min**	**Max**
hsa-miR-1271-5p	4.4791036	4.2760125	1.892052	6.561436
hsa-miR-96-5p	1.99137435	1.5174105	0.6139191	5.378518
**Family**	**miR-99ab/100**			
**2/2**	**Mean avg log2 signal**	**Median avg log2 signal**	**Min**	**Max**
hsa-miR-100-5p	6.1346097	6.400215	1.58824	10.17722
hsa-miR-99a-5p	4.25628325	4.2469215	1.614767	7.333538
hsa-miR-99b-5p	5.8403383	5.5560085	1.841449	8.240961
**FAMILIES IN SERUM**				
**Family**	**let-7/98/4458/4500**			
**5/11**	**Mean avg log2 signal**	**Median avg log2 signal**	**Min**	**Max**
hsa-let-7a-5p	2.35574122	1.3626195	0.9671984	8.522055
hsa-let-7b-5p	8.722545235	11.001215	0.9136667	14.10612
hsa-let-7c-5p	4.2065813	2.4261785	0.8663921	13.40558
hsa-let-7d-5p	3.065713295	1.77158	0.8507624	10.76772
hsa-let-7i-5p	4.007933925	2.499583	0.8028431	11.47828
**Family**	**miR-103a/107/107ab**			
**2/2**	**Mean avg log2 signal**	**Median avg log2 signal**	**Min**	**Max**
hsa-miR-103a-3p	5.44771043	4.5605605	0.8645353	12.70128
hsa-miR-107	5.73106166	5.388365	0.7056093	12.39119
**Family**	**miR-1207-5p/4763-3p**			
**2/2**	**Mean avg log2 signal**	**Median avg log2 signal**	**Min**	**Max**
hsa-miR-1207-5p	6.03900415	5.420463	1.270911	12.58873
hsa-miR-4763-3p	13.020282	13.21241	11.24157	13.86009
**Family**	**miR-1260/1260b/1391**			
**2/2**	**Mean avg log2 signal**	**Median avg log2 signal**	**Min**	**Max**
hsa-miR-1260a	2.666001305	2.206878	0.8479171	6.457009
hsa-miR-1260b	3.5746712	3.0856235	1.075187	9.426715
**Family**	**miR-1268/1268b**			
**2/2**	**Mean avg log2 signal**	**Median avg log2 signal**	**Min**	**Max**
hsa-miR-1268a	7.084842	8.0149515	1.21516	13.26256

hsa-miR-1268b	5.4117592	3.2747305	1.043564	13.25067
**Family**	**miR-130ac/301ab/301b/301b-3p/454/721/4295/3666**			
**2/4**	**Mean avg log2 signal**	**Median avg log2 signal**	**Min**	**Max**
hsa-miR-130a-3p	2.118834955	1.371458	0.6623483	8.970987
hsa-miR-130b-3p	3.81855781	2.008955	0.8781023	12.1135
**Family**	**miR-1346/3940-5p/4507**			
**2/2**	**Mean avg log2 signal**	**Median avg log2 signal**	**Min**	**Max**
hsa-miR-3940-5p	12.6052633	12.997545	8.817486	14.00869
hsa-miR-4507	12.0132295	12.524135	10.07962	13.2831
**Family**	**miR-15abc/16/16abc/195/322/424/497/1907**			
**3/6**	**Mean avg log2 signal**	**Median avg log2 signal**	**Min**	**Max**
hsa-miR-15a-5p	1.42684183	1.279123	0.8413577	3.226015
hsa-miR-15b-5p	2.082156905	1.2515565	0.8274632	9.97176
hsa-miR-16-5p	8.148942835	11.60187	0.7741661	14.16446
**Family**	**miR-17/17-5p/20ab/20b-5p/93/106ab/427/518a-3p/519d**			
**6/7**	**Mean avg log2 signal**	**Median avg log2 signal**	**Min**	**Max**
hsa-miR-106a-5p	5.3289351	2.530807	1.188536	12.88918
hsa-miR-106b-5p	5.566443385	5.748272	0.8753357	11.94596
hsa-miR-17-5p	6.591437085	7.9273075	0.6702414	13.05287
hsa-miR-20a-5p	4.16534026	2.135872	0.7367697	12.22935
hsa-miR-20b-5p	2.54532543	1.21408	0.761919	8.38322
hsa-miR-93-5p	7.44756748	9.915284	0.8739204	13.30775
**Family**	**miR-181abcd/4262**			
**2/5**	**Mean avg log2 signal**	**Median avg log2 signal**	**Min**	**Max**
hsa-miR-181a-5p	3.03341475	1.560447	1.040275	9.706189
hsa-miR-181b-5p	1.57859475	1.4216735	0.7873859	3.882611
**Family**	**miR-185/882/3473/4306/4644**			
**2/2**	**Mean avg log2 signal**	**Median avg log2 signal**	**Min**	**Max**
hsa-miR-185-5p	10.10144385	12.752095	1.352456	13.71814
hsa-miR-4306	2.016822855	1.358952	0.7833662	7.626094
**Family**	**miR-221/222/222ab/1928**			
**2/2**	**Mean avg log2 signal**	**Median avg log2 signal**	**Min**	**Max**
hsa-miR-3940-5p	12.6052633	2.537273	0.6532183	11.25889
hsa-miR-4507	12.0132295	1.637232	0.884634	10.15192
**Family**	**miR-23abc/23b-3p**			
**3/3**	**Mean avg log2 signal**	**Median avg log2 signal**	**Min**	**Max**
hsa-miR-23a-3p	8.055227245	11.66187	0.9300919	13.43877
hsa-miR-23b-3p	4.240516745	2.0237855	0.7400599	10.50028
hsa-miR-23c	1.71279453	1.516901	0.9382154	4.21056
**Family**	**miR-25/32/92abc/363/363-3p/367**			
**4/6**	**Mean avg log2 signal**	**Median avg log2 signal**	**Min**	**Max**

hsa-miR-25-3p	7.151478735	9.6425435	0.7854748	13.13439
hsa-miR-32-5p	2.09611176	1.77058	0.9258198	8.630038
hsa-miR-92a-3p	10.36045135	13.43241	1.34765	14.31478
hsa-miR-92b-3p	5.373205925	5.3916845	0.9702005	11.65615
**Family**	**miR-30abcdef/30abe-5p/384-5p**			
**2/5**	**Mean avg log2 signal**	**Median avg log2 signal**	**Min**	**Max**
hsa-miR-30a-5p	1.536562725	1.3223155	0.7598438	5.327148
hsa-miR-30d-5p	2.7577055	1.934687	1.10609	6.025137
**Family**	**miR-320abcd/4429**			
**5/5**	**Mean avg log2 signal**	**Median avg log2 signal**	**Min**	**Max**
hsa-miR-320a	13.527687	13.62571	12.43165	14.04206
hsa-miR-320b	13.436803	13.56419	12.24199	13.92185
hsa-miR-320c	13.282577	13.46865	12.13927	13.82717
hsa-miR-320d	11.975554	12.15997	94.82784	13.34144
hsa-miR-4429	8.517073	9.414505	0.866147	12.65761
**Family**	**miR-3201/4791**			
**2/2**	**Mean avg log2 signal**	**Median avg log2 signal**	**Min**	**Max**
hsa-miR-3201	4.7130031	1.8166565	1.062672	13.54973
hsa-miR-4791	1.482768505	1.158821	0.5851555	4.649663
**Family**	**miR-378/422a/378bcdefhi**			
**5/9**	**Mean avg log2 signal**	**Median avg log2 signal**	**Min**	**Max**
hsa-miR-378a-3p	3.776996615	2.1639765	0.9258423	11.02324
hsa-miR-378c	2.137942045	1.6998945	0.7367697	6.024651
hsa-miR-378f	1.613916685	1.25083	0.8604646	6.608524
hsa-miR-378h	8.5265404	8.4223635	2.201951	12.75222
hsa-miR-378i	1.5043903	1.1657165	0.6607714	6.923134
**Family**	**miR-4481/4745-5p**			
**2/2**	**Mean avg log2 signal**	**Median avg log2 signal**	**Min**	**Max**
hsa-miR-4481	1.97176223	1.7406065	0.8108046	5.396856
hsa-miR-4745-5p	11.8925181	13.07332	2.480342	14.06308
**Family**	**miR-548abakhjiwy/548abcd-5p/559**			
**2/12**	**Mean avg log2 signal**	**Median avg log2 signal**	**Min**	**Max**
hsa-miR-548i	1.03648826	1.02679345	0.5986478	1.846887
hsa-miR-559	1.50460346	1.306624	0.849665	3.120622
**Family**	**miR-548aeajamx**			
**3/4**	**Mean avg log2 signal**	**Median avg log2 signal**	**Min**	**Max**
hsa-miR-548ae-3p	4.02563842	3.8453635	0.9625864	8.106996
hsa-miR-548aj-3p	2.621365225	1.864342	0.9912795	7.328541
hsa-miR-548x-3p	2.9407676	1.878434	1.070633	7.826774

Family<br><br>2/4	miR-548d-3p/548acbz			
	Mean avg log2 signal	Median avg log2 signal	Min	Max
hsa-miR-548ac	4.4635448	3.4339315	1.070057	10.00958
hsa-miR-548z	2.12570635	1.5744575	1.016919	6.564924

Family<br><br>2/3	miR-762/4492/4498			
	Mean avg log2 signal	Median avg log2 signal	Min	Max
hsa-miR-4492	4.76235322	3.190722	0.9150324	12.83638
hsa-miR-762	13.3660885	13.57404	11.78227	14.20542

Table S4: miR-378h target gene enrichment analysis

PANTHER Pathways	H. sapiens (RFF) #	upload_1 #	expected	Fold Enrichment	raw P value	FDR
Glutamine glutamate conversion	4	1	.01	> 100	8.04E-03	2.62E-01
Hypoxia response via HIF activation	33	2	.05	37.51	1.45E-03	2.37E-01
FAS signaling pathway	34	2	.05	36.40	1.54E-03	1.25E-01
p53 pathway by glucose deprivation	23	1	.04	26.91	3.80E-02	4.77E-01
VEGF signaling pathway	68	2	.11	18.20	5.70E-03	3.09E-01
Insulin/IGF pathway-protein kinase B signaling cascade	41	1	.07	15.09	6.56E-02	7.64E-01
p53 pathway feedback loops 2	51	1	.08	12.13	8.06E-02	8.76E-01
PI3 kinase pathway	55	1	.09	11.25	8.66E-02	8.82E-01
Oxidative stress response	58	1	.09	10.67	9.10E-02	8.72E-01
Muscarinic acetylcholine receptor 2 and 4 signaling pathway	63	1	.10	9.82	9.83E-02	8.90E-01
Heterotrimeric G-protein signaling pathway-Gq alpha and Go alpha mediated pathway	126	2	.20	9.82	1.81E-02	4.91E-01
EGF receptor signaling pathway	140	2	.23	8.84	2.20E-02	5.11E-01
Huntington disease	145	2	.23	8.54	2.34E-02	4.77E-01
Gonadotropin-releasing hormone receptor pathway	237	3	.38	7.83	6.73E-03	2.74E-01
Heterotrimeric G-prot sig. pathway-Gi alpha and Gs alpha mediated pathway	164	2	.26	7.55	2.93E-02	5.31E-01
Angiogenesis	173	2	.28	7.15	3.23E-02	4.79E-01
CCKR signaling map	174	2	.28	7.11	3.27E-02	4.44E-01

Table S5. Differently expressed miRNA between HCC and Normal Tissue from StarBase v3.0 Database

miRNA transcript	CancerExp log2(RPM+0.01)	NormalExp log2(RPM+0.01)	FoldChange	pValue	FDR
let-7a-5p	30211.46	36668.13	0.82	0.00033	0.0022
let-7b-5p	7507.67	12249.94	0.61	4.60E-11	1.20E-09
let-7c-5p	2230.23	5606.98	0.4	3.90E-20	3.90E-18
let-7d-5p	204.08	167.29	1.22	ns	ns
let-7f-1-3p	2.29	3.27	0.7	9.70E-05	0.00074
let-7i-5p	227.98	232.63	0.98	ns	ns
miR-103a-3p	30968.92	16382.16	1.89	9.30E-13	3.10E-11
miR-106a-5p	11.8	9.91	1.19	0.04	ns
miR-106b-5p	292.78	164.4	1.78	3.90E-09	6.90E-08
miR-107	151.54	116.53	1.3	0.013	0.053
miR-1184	nd				
miR-1207-5p	nd				
miR-1224-5p	0.9	0.21	4.26	ns	ns
miR-1225-5p	nd				
miR-122-5p	23817.07	38890.1	0.61	6.90E-06	6.60E-05
miR-1228-3p	1.29	1.58	0.81	0.0018	0.0098
miR-1234-3p	0.08	0.06	1.36	ns	ns
miR-1255a	0.09	0.11	0.81	ns	ns
miR-125b-5p	569.76	1201.99	0.47	2.00E-18	1.60E-16
miR-1260b	0.06	0.03	1.88	ns	ns
miR-126-3p	5175.46	7403.48	0.7	1.10E-08	1.90E-07
miR-1268b	nd				
miR-1280	nd				
miR-1281	nd				
miR-1307-3p	1757.38	862.08	2.04	4.80E-11	1.20E-09
miR-130a-3p	54.12	119.94	0.45	5.20E-19	4.50E-17
miR-130b-3p	32.41	15.49	2.09	0.00012	0.00086
miR-140-3p	1693.46	1114.73	1.52	8.40E-12	2.40E-10
miR-143-3p	49951.54	60011.86	0.83	0.00087	0.0053
miR-145-5p	1079.12	2310.46	0.47	7.20E-16	3.60E-14
miR-1469	0.01	0.01	1	ns	ns
miR-150-5p	466.3	749.86	0.62	1.60E-06	1.80E-05
miR-151a-3p	2777.42	1893.57	1.47	0.0024	0.013
miR-151a-5p	163.7	104.19	1.57	3.00E-06	3.10E-05
miR-155-5p	278.49	206.78	1.35	ns	ns
miR-1587	nd				
miR-15a-5p	233.93	170.76	1.37	0.0011	0.0066
miR-15b-5p	252.64	186.43	1.36	ns	ns
miR-16-5p	472.39	583.1	0.81	7.00E-05	0.00054

miR-17-5p	932.86	447.88	2.08	1.90E-05	0.00017
miR-181a-5p	1109.61	807.06	1.37	0.044	ns
miR-181b-5p	239.39	136.4	1.76	0.00091	0.0055
miR-1825	0.03	0.05	0.64	ns	ns
miR-18a-5p	26.51	8.51	3.12	2.50E-05	0.00022
miR-1908-5p	0.03	0.04	0.88	ns	ns
miR-1909-5p	0.02	0.02	0.95	ns	ns
miR-1910-5p	0.05	0.03	1.54	ns	ns
miR-191-3p	1.47	2.62	0.56	1.70E-06	1.80E-05
miR-1915-5p	0.12	0.04	2.95	0.025	ns
miR-191-5p	614.4	704.89	0.87	0.002	0.011
miR-193a-5p	586.06	730.95	0.8	1.70E-05	0.00015
miR-197-3p	369.27	382.92	0.96	0.047	ns
miR-202-5p	0.46	0.22	2.07	ns	ns
miR-20a-5p	803.78	365.63	2.2	3.70E-07	4.60E-06
miR-20b-5p	22.5	8.99	2.5	0.026	ns
miR-2116-3p	0.63	0.67	0.94	0.56	0.78
miR-221-3p	143.41	56.12	2.56	1.90E-13	6.70E-12
miR-222-3p	34.22	13.79	2.48	1.50E-11	4.20E-10
miR-223-3p	203.87	266.41	0.77	2.50E-07	3.20E-06
miR-2277-5p	0.7	0.49	1.42	ns	ns
miR-23a-3p	1547.08	1529.14	1.01	ns	ns
miR-23b-3p	1575.05	2037.4	0.77	9.00E-07	1.00E-05
miR-25-3p	10690.02	6366.1	1.68	7.00E-11	1.60E-09
miR-26a-5p	2217.95	4100.48	0.54	1.70E-22	2.10E-20
miR-27a-3p	674.21	723.1	0.93	0.012	0.049
miR-2861	nd				
miR-29a-3p	7378.83	12128.88	0.61	2.30E-12	7.30E-11
miR-30a-5p	18903.43	22251.94	0.85	0.00014	0.001
miR-30d-5p	15899.47	7954.91	2	1.50E-08	2.60E-07
miR-3121-3p	0.03	0.02	1.28	ns	ns
miR-3135b	nd				
miR-3141	nd				
miR-3153	nd				
miR-3157-3p	0.31	0.47	0.66	0.0023	0.012
miR-3162-3p	0.02	0.02	1.19	ns	ns
miR-3162-5p	0.03	0.01	2.52	ns	ns
miR-3178	0.01	0.02	0.9	ns	ns
miR-3196	0.03	0.02	1.28	ns	ns
miR-3197	nd				
miR-320e	0.06	0.09	0.68	0.014	ns
miR-323b-3p	9.55	4.07	2.35	0.005	0.024
miR-337-3p	55.42	56.4	0.98	1.60E-08	2.70E-07
miR-342-3p	105.08	108.99	0.96	0.05	ns
miR-3613-3p	0.73	0.94	0.78	0.021	ns
miR-3613-5p	16.25	13.89	1.17	ns	ns

miR-361-5p	347.94	271.42	1.28	0.0048	0.023
miR-3619-5p	0.04	0.03	1.6	ns	ns
miR-3646	0.02	0.01	1.11	ns	ns
miR-3656	0.01	0.03	0.49	0.00028	0.0019
miR-3663-3p	0.02	0.01	1.69	ns	ns
miR-3679-5p	0.07	0.05	1.37	ns	ns
miR-371b-5p	0.03	0.04	0.81	ns	ns
miR-378a-3p	1265.16	2268.86	0.56	7.90E-12	2.30E-10
miR-378c	18.9	38.24	0.49	1.90E-15	8.90E-14
miR-378f	0.06	0.11	0.56	0.0005	0.0032
miR-378h	nd				
miR-378i	0.16	0.34	0.46	2.70E-05	0.00023
miR-3921	nd				
miR-3935	nd				
miR-3940-5p	0.47	0.46	1.03	ns	ns
miR-3942-3p	0.07	0.06	1.16	ns	ns
miR-409-3p	39.41	19.38	2.03	ns	ns
miR-423-5p	69.46	40.23	1.73	4.00E-12	1.20E-10
miR-425-5p	208.62	116	1.8	1.30E-05	0.00012
miR-4258	nd				
miR-4274	nd				
miR-4281	nd				
miR-4286	0.18	0.32	0.56	1.80E-06	1.90E-05
miR-4290	nd				
miR-4306	0.04	0.05	0.85	ns	ns
miR-4310	nd				
miR-4429	nd				
miR-4430	0.02	0.04	0.5	0.0079	0.035
miR-4433a-3p	nd				
miR-4443	0.34	0.7	0.49	2.00E-07	2.70E-06
miR-4452	nd				
miR-4454	0.18	0.41	0.44	2.80E-07	3.50E-06
miR-4463	nd				
miR-4466	0.05	0.07	0.78	ns	ns
miR-4481	nd				
miR-4487	0.02	0.03	0.8	ns	ns
miR-4488	0.03	0.05	0.56	0.0083	0.037
miR-4492	nd				
miR-4497	nd				
miR-4505	0.03	0.05	0.56	0.015	0.061
miR-4507	nd				
miR-450b-5p	13.85	27.69	0.5	7.30E-17	4.50E-15
mir-451-3p	886.23	1900.63	0.47	1.00E-16	5.70E-15
miR-4514	nd				
miR-4516	0.02	0.01	1.79	ns	ns
miR-4529-3p	0.06	0.08	0.81	ns	ns

miR-4530	nd				
miR-4532	0.01	0.01	0.86	ns	ns
miR-4539	0.01	0.01	1.03	ns	ns
miR-455-3p	756.52	1541.15	0.49	1.40E-16	7.70E-15
miR-4646-3p	0.04	0.06	0.68	ns	ns
miR-4652-3p	nd				
miR-4668-5p	nd				
miR-4674	0.16	0.13	1.17	ns	ns
miR-4690-5p	0.04	0.05	0.82	ns	ns
miR-4701-5p	0.04	0.03	1.46	ns	ns
miR-4706	0.02	0.01	1.7	ns	ns
miR-4707-5p	0.09	0.07	1.41	ns	ns
miR-4708-5p	0.01	0.01	1.12	ns	ns
miR-4722-3p	0.01	0.01	1.11	ns	ns
miR-4725-5p	0.04	0.02	1.86	ns	ns
miR-4741	0.19	0.08	2.36	0.014	ns
miR-4745-5p	0.07	0.03	2.1	ns	ns
miR-4763-3p	nd				
miR-4789-5p	nd				
miR-4793-3p	0.05	0.02	2.79	0.028	ns
miR-4800-3p	0.09	0.36	0.24	2.50E-14	9.90E-13
miR-486-3p	0.42	0.82	0.52	1.30E-08	2.30E-07
miR-486-5p	203.92	336.09	0.61	5.10E-14	1.90E-12
miR-491-5p	1.45	1.64	0.89	0.016	ns
miR-502-3p	14.26	8.15	1.75	4.00E-08	6.30E-07
miR-503-5p	11.44	8.57	1.33	ns	ns
miR-532-5p	1621.16	702.65	2.31	4.10E-16	2.10E-14
miR-548a-3p	nd				
miR-548ac	nd				
miR-548ae-3p	nd				
miR-548u	nd				
miR-550a-3p	2.33	1.93	1.21	0.76	0.82
miR-559	nd				
miR-563	nd				
miR-570-3p	1.27	1.11	1.14	ns	ns
miR-574-3p	135.05	209.16	0.65	1.70E-11	4.70E-10
miR-574-5p	nd				
miR-584-5p	53.68	45.03	1.19	ns	ns
miR-602	nd				
miR-629-3p	2.92	2.62	1.11	ns	ns
miR-635	nd				
miR-638	nd				
miR-663a	nd				
miR-664a-5p	5.26	4.66	1.13	ns	ns
miR-762	nd				
miR-877-3p	0.17	0.12	1.45	ns	ns

miR-885-3p	4.99	5.49	0.91	0.0019	0.011
miR-885-5p	407.2	467.06	0.87	0.0016	0.0088
miR-92a-3p	23597.42	16906.15	1.4	ns	ns
miR-92b-3p	20.41	10.8	1.89	ns	ns
miR-933	0.02	0.02	1.24	ns	ns
miR-93-5p	6759.66	2313.62	2.92	6.80E-26	1.50E-23
miR-940	0.4	0.74	0.55	5.60E-07	6.60E-06
miR-940	1.89	1.2	1.58	ns	ns

SUPPLEMENTARY FIGURES

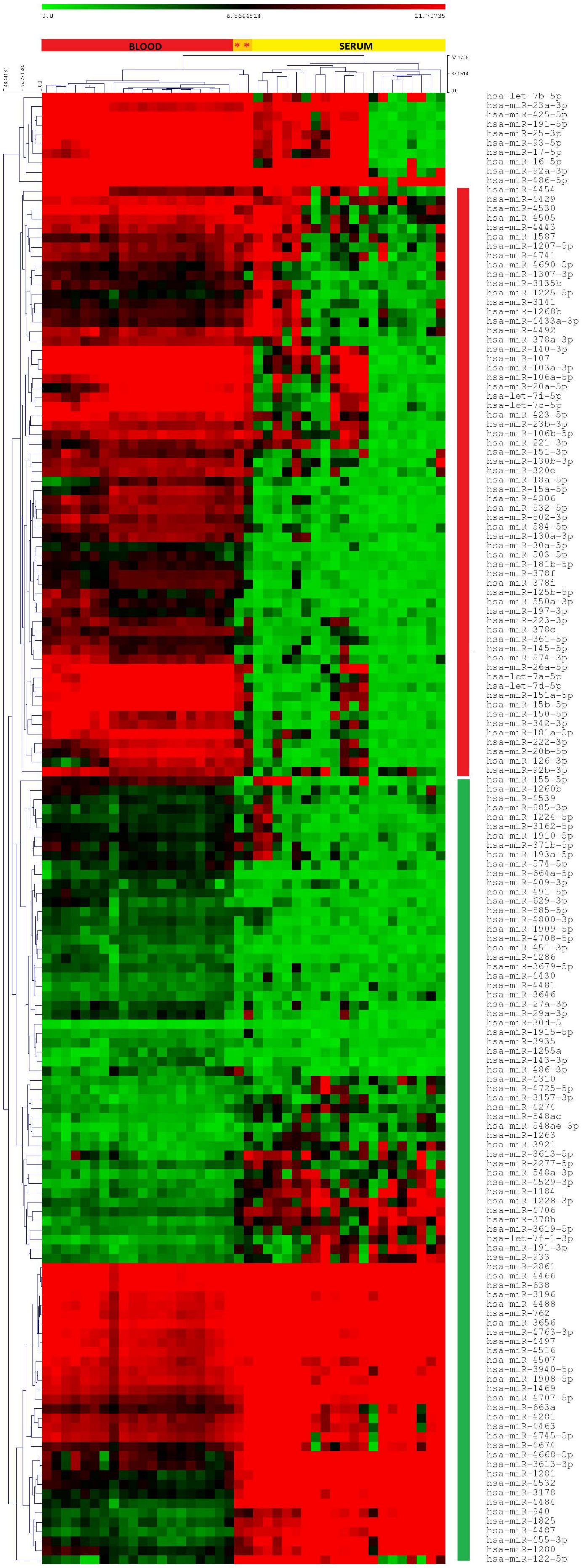

Figure S1. Heatmap with the pseudocolor scale underneath, of the differentially expressed miRNAs between serum and blood. The heatmap includes two haemolyzed serum samples (Hb 63.54 and 379 mg/mL), marked with red asterisks. Unsupervised hierarchical clustering is used to order samples and miRNAs, the log2-transformed microarray signal was considered. The sample tree with optimized leaf-ordering is drawn using Euclidean distances and average linkages for cluster-to-cluster distance. The red cluster evidenced the miRNAs influenced by hemolysis, the green cluster the unaffected ones.

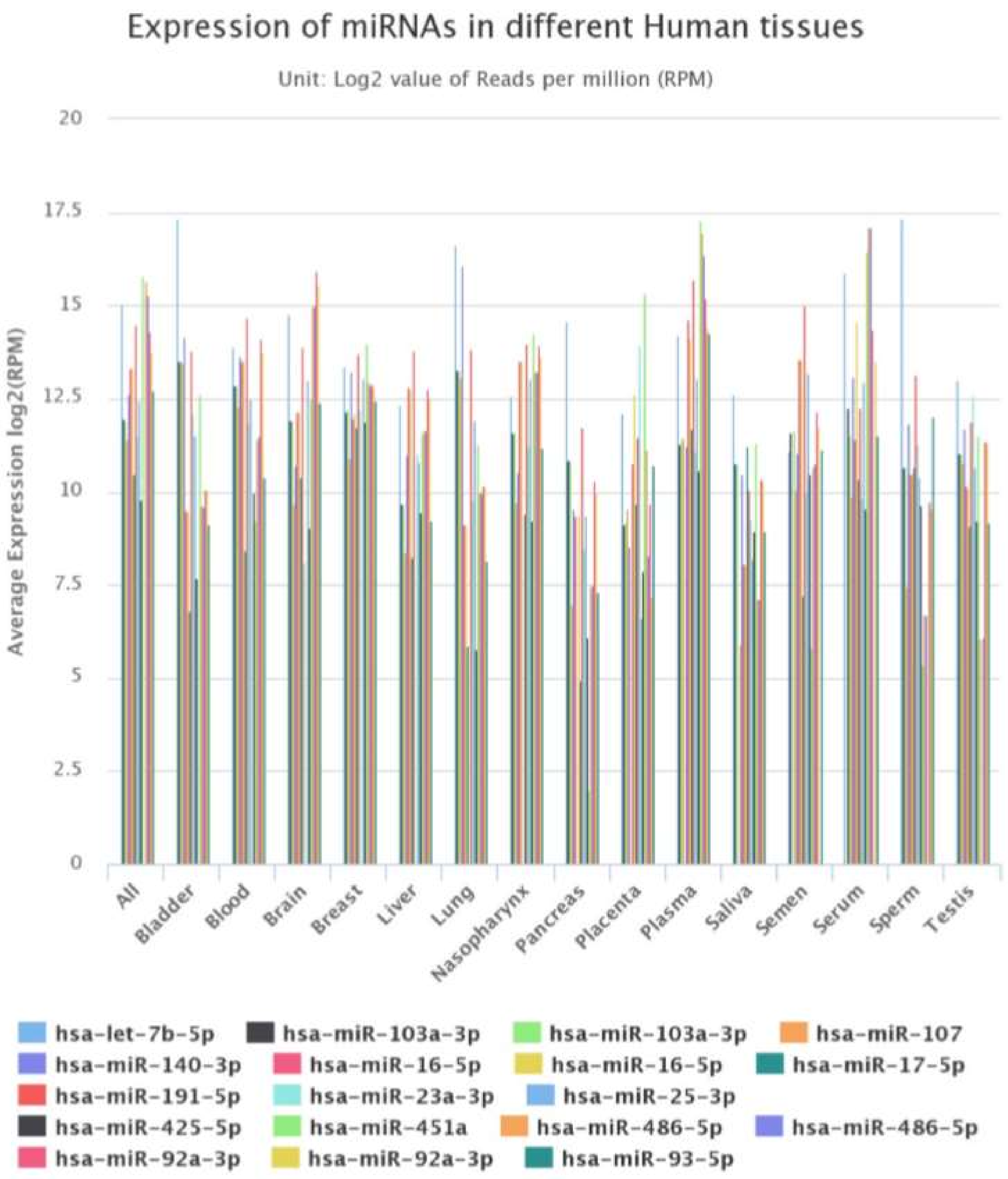

Figure S2. MiRNAs from cluster 1 are widely expressed among different human tissues at high levels. Source: http://guanlab.ccmb.med.umich.edu/mirmine/

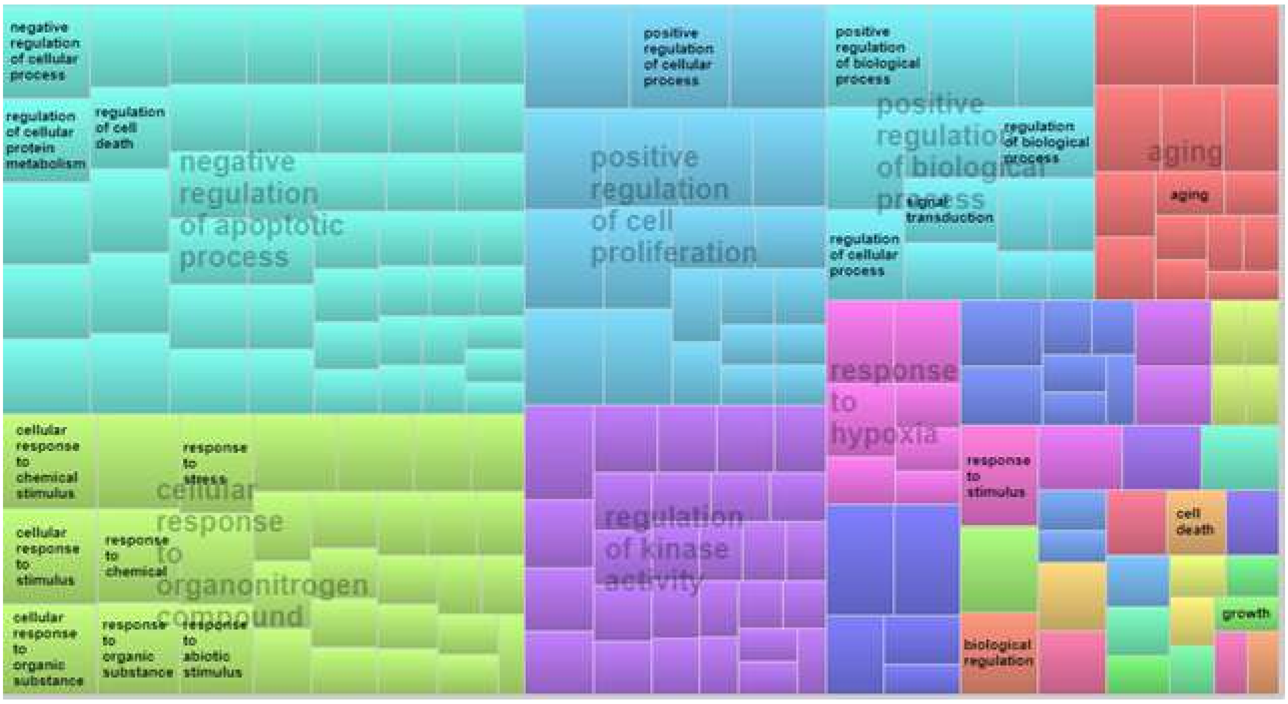

Figure S3. GO enriched terms treeMap of the 433 validated target miRNAs from cluster 1. The size of the rectangles is proportional to the adjusted p-value of the enriched GO term relative to the background list.

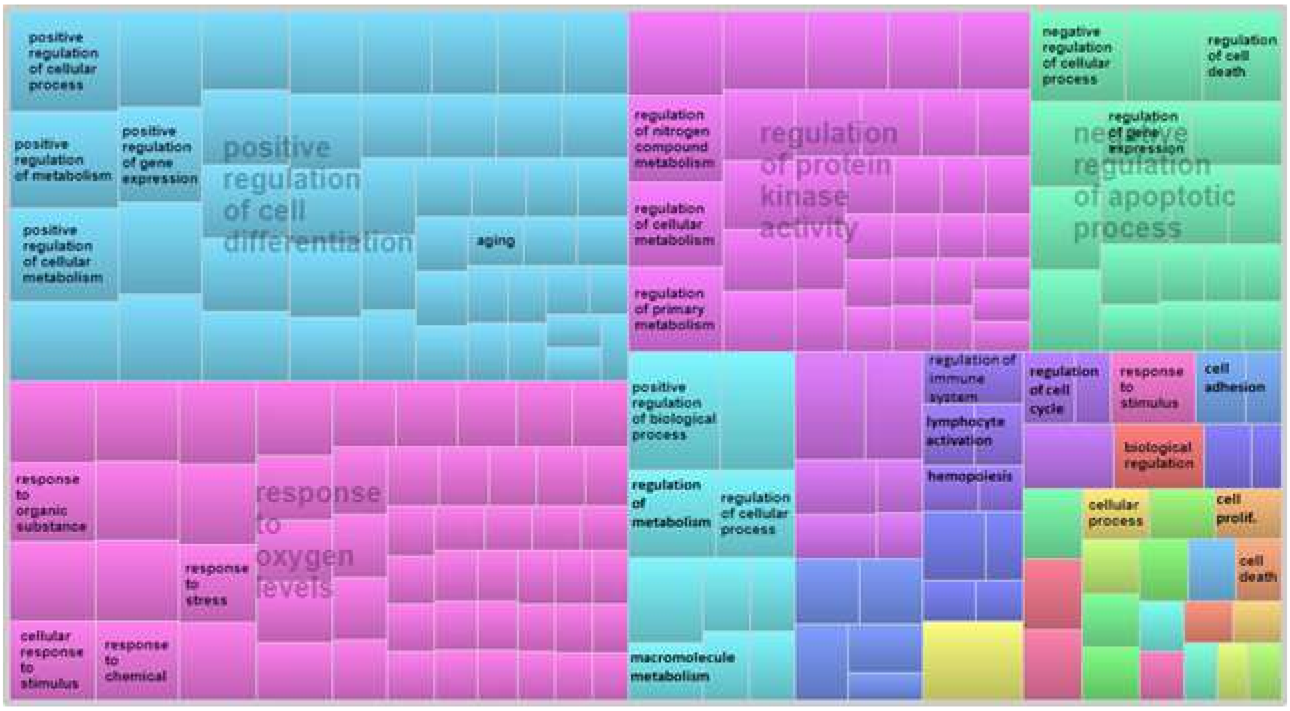

Figure S4. GO enriched terms treeMap of the validated target miRNAs from cluster 2. The size of the rectangles is proportional to the adjusted p-value of the enriched GO term relative to the background list.

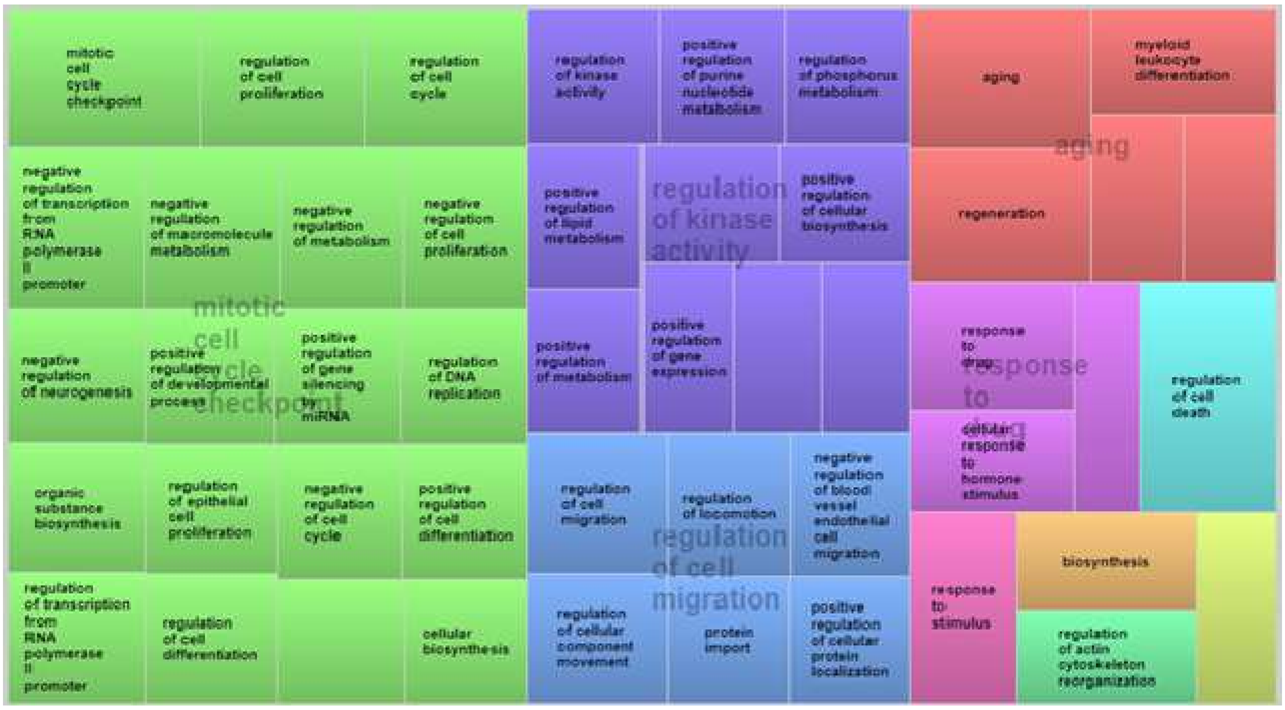

Figure S5. GO enriched terms treeMap of the validated target miRNAs from cluster 5. The size of the rectangles is proportional to the adjusted p-value of the enriched GO term relative to the background list.

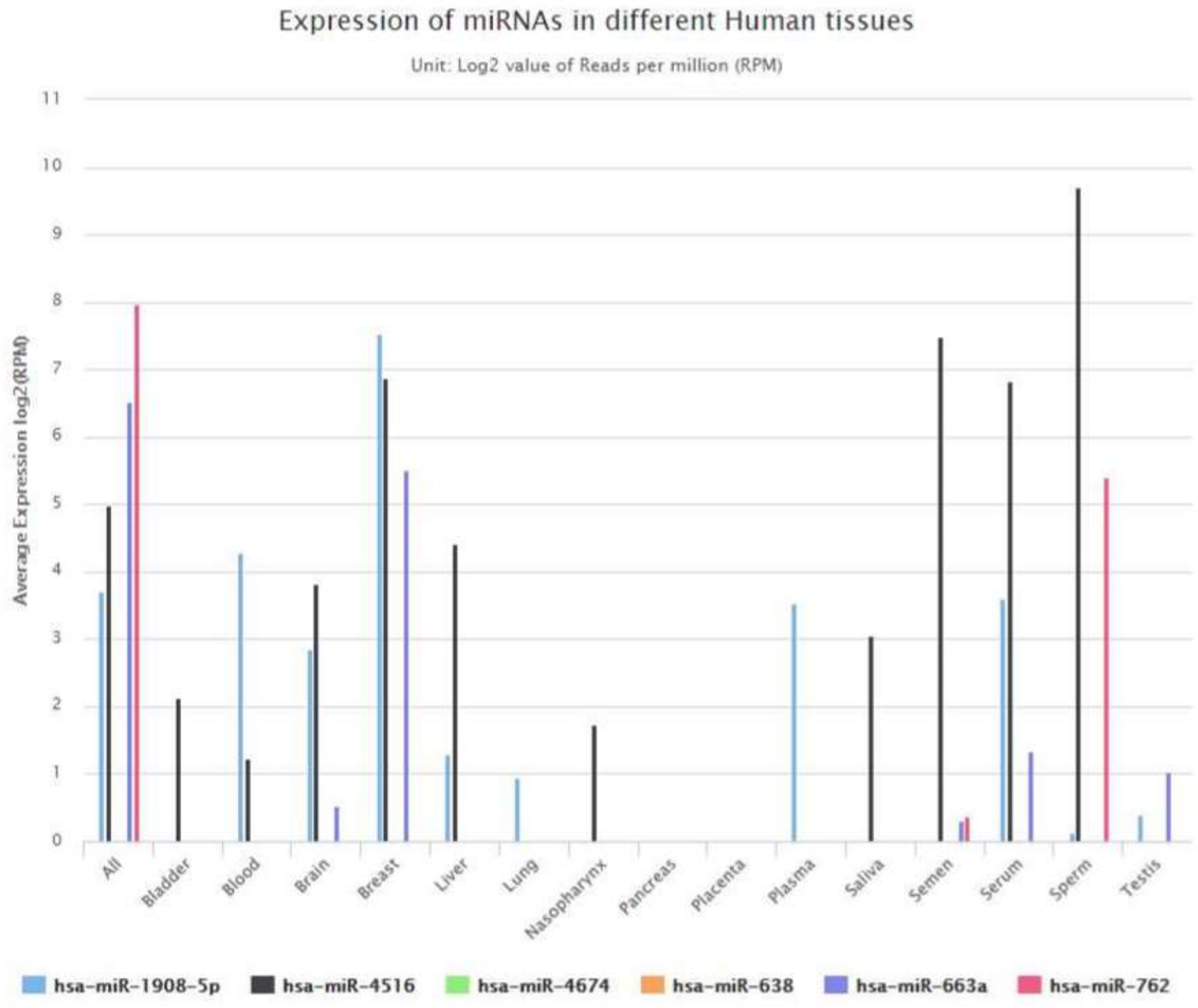

Figure S6. Limited information exist about the expression profiles of the miRNAs from cluster 5. Source: http://guanlab.ccmb.med.umich.edu/mirmine/

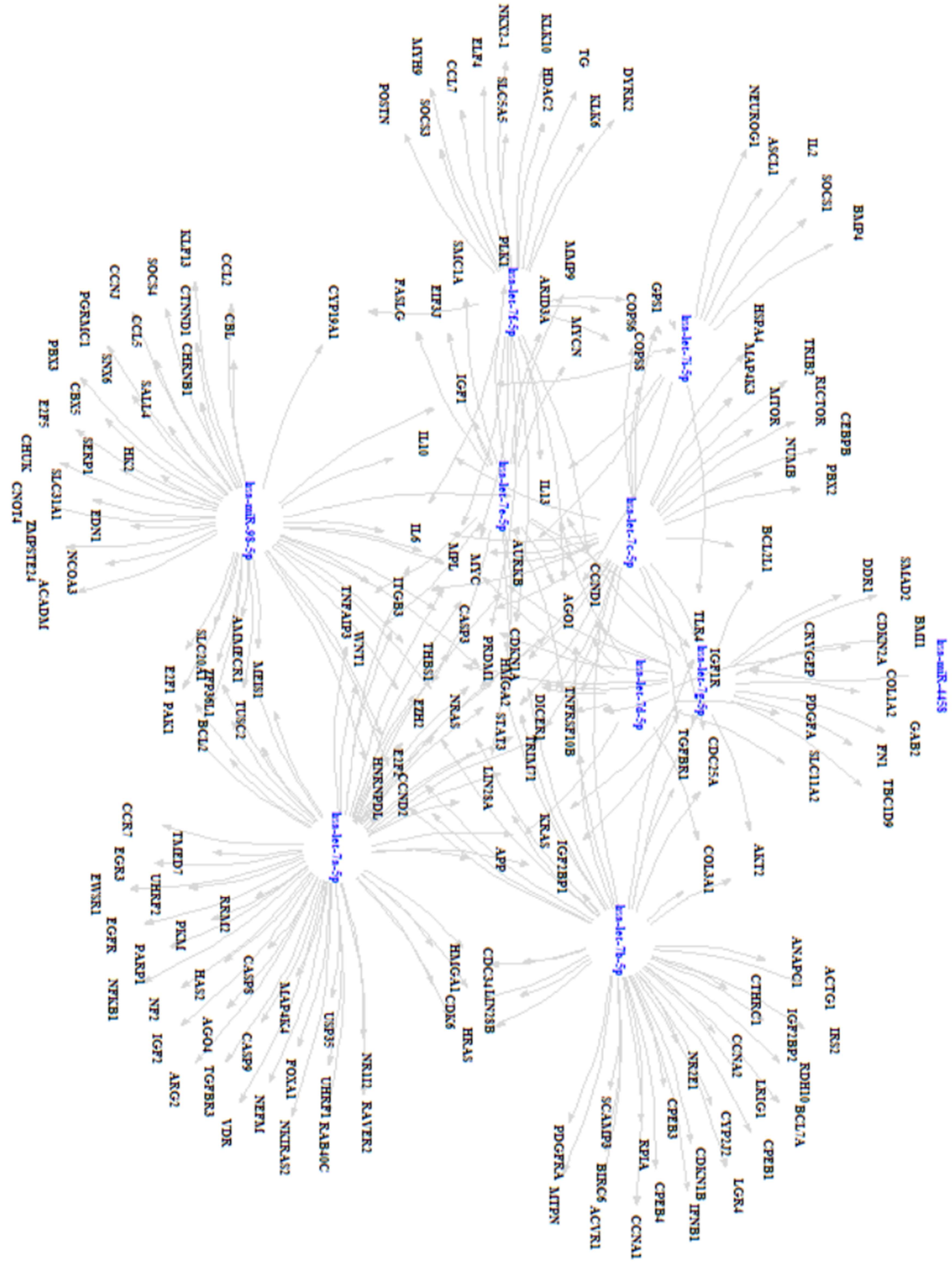

Figure S7. Let7 family validated target network built with Tidyverse (version 1.2.1) and Igraph (version 1.1.2) packages in R environment. Validated targets were downloaded from miRTarBase. Targets with strong experimental evidences were considered.

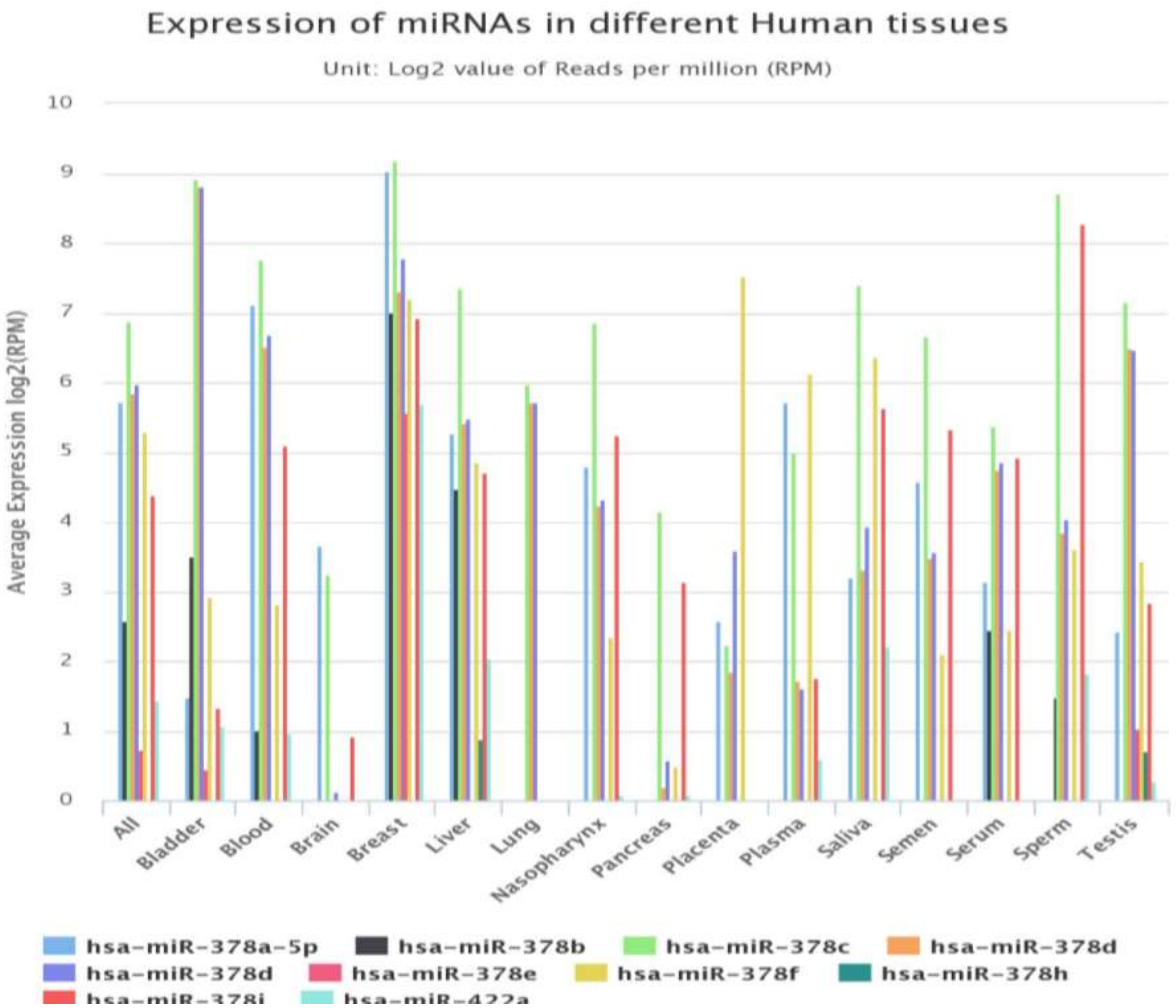

Figure S8. Mir-378 family expression patter in different tissues. Mir-378h was detected in liver and testis only. Source: http://guanlab.ccmb.med.umich.edu/mirmine/
